# Comparative Metagenomics Provides Insight Into the Ecosystem Functioning of the Shark Bay Stromatolites, Western Australia

**DOI:** 10.3389/fmicb.2018.01359

**Published:** 2018-06-25

**Authors:** Joany Babilonia, Ana Conesa, Giorgio Casaburi, Cecile Pereira, Artemis S. Louyakis, R. Pamela Reid, Jamie S. Foster

**Affiliations:** ^1^Space Life Science Lab, Department of Microbiology and Cell Science, University of Florida, Gainesville, FL, United States; ^2^Department of Microbiology and Cell Science, Genetics Institute, Institute for Food and Agricultural Sciences, University of Florida, Gainesville, FL, United States; ^3^Genomics of Gene Expression Laboratory, Prince Felipe Research Center, Valencia, Spain; ^4^EURA NOVA, Marseille, France; ^5^Rosenstiel School of Marine and Atmospheric Science, University of Miami, Miami, FL, United States

**Keywords:** stromatolite, microbial mat, comparative metagenomics, Hamelin Pool, Shark Bay

## Abstract

Stromatolites are organosedimentary build-ups that have formed as a result of the sediment trapping, binding and precipitating activities of microbes. Today, extant systems provide an ideal platform for understanding the structure, composition, and interactions between stromatolite-forming microbial communities and their respective environments. In this study, we compared the metagenomes of three prevalent stromatolite-forming microbial mat types in the Spaven Province of Hamelin Pool, Shark Bay located in Western Australia. These stromatolite-forming mat types included an intertidal pustular mat as well as a smooth and colloform mat types located in the subtidal zone. Additionally, the metagenomes of an adjacent, non-lithifying mat located in the upper intertidal zone were also sequenced for comparative purposes. Taxonomic and functional gene analyses revealed distinctive differences between the lithifying and non-lithifying mat types, which strongly correlated with water depth. Three distinct populations emerged including the upper intertidal non-lithifying mats, the intertidal pustular mats associated with unlaminated carbonate build-ups, and the subtidal colloform and smooth mat types associated with laminated structures. Functional analysis of metagenomes revealed that amongst stromatolite-forming mats there was an enrichment of photosynthesis pathways in the pustular stromatolite-forming mats. In the colloform and smooth stromatolite-forming mats, however, there was an increase in the abundance of genes associated with those heterotrophic metabolisms typically associated with carbonate mineralization, such as sulfate reduction. The comparative metagenomic analyses suggest that stromatolites of Hamelin Pool may form by two distinctive processes that are highly dependent on water depth. These results provide key insight into the potential adaptive strategies and synergistic interactions between microbes and their environments that may lead to stromatolite formation and accretion.

## Introduction

Stromatolites are one of the most prevalent and recognizable components of the fossil record, dating back more than 3.7 Ga years (Grotzinger and Knoll, 1999; Nutman et al., 2016). These lithified, organosedimentary structures are a type of microbialite, formed by the sediment trapping, binding and/or carbonate precipitating activities of microorganisms in response to their local environment (Awramik et al., 1976; Burne and Moore, 1987). Stromatolites are distinguished from other microbialites by the presence of laminated microstructures, which are formed through iterative successions of sedimentation, microbial mat growth, and lithification (Reid et al., 2000; Dupraz and Visscher, 2005; Pace et al., 2018). These long-lived microbialite ecosystems have had a profound impact on the habitability of the planet, as they are attributed to changing the global redox conditions via oxygenic photosynthesis (Des Marais, 1991; Lyons et al., 2014). These properties make contemporary microbialites ideal model systems for investigating carbon cycling and the underlying processes associated with the precipitation and dissolution of calcium carbonate (Riding, 2000; Dupraz and Visscher, 2005; ?).

Modern microbialite ecosystems are widespread and are found in a diverse range of environments including, but not limited to, lacustrine ecosystems (e.g., Cuatro Ciénegas, Mexico; Lake Alchichica, Mexico; Pavilion and Kelly Lake, British Columbia) (Osborne et al., 1982; Ferris et al., 1997; Laval et al., 2000; Souza et al., 2006; Couradeau et al., 2011; Theisen et al., 2015; Chagas et al., 2016; White et al., 2016), freshwater systems (e.g., Deer Cave and Giblin River; Lundberg and McFarlane, 2011; Proemse et al., 2017); geothermal springs (e.g., Yellowstone National Park, WY, United States) (Inskeep et al., 2004; Pepe-Ranney et al., 2012), open marine environments (e.g., Exuma Sound, The Bahamas) (Dravis, 1983; Dill et al., 1986; Reid et al., 2000; Khodadad and Foster, 2012), and hypersaline waters (e.g., Kiritimati Atoll, Great Salt Lake) (Burns et al., 2004; Schneider et al., 2013; Ruvindy et al., 2016; Suosaari et al., 2016a; Lindsay et al., 2017).

**FIGURE 1:**
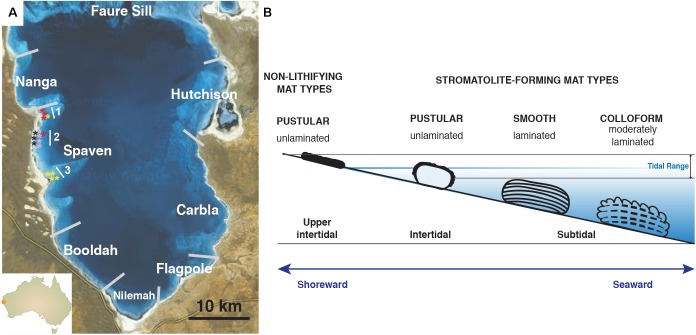
Overview of Hamelin Pool collection site and locations of stromatolite-forming and non-lithifying mat types within the pool. **(A)** Map of Provinces located within Hamelin Pool, Shark Bay. The stars reflect the collection sites of the colloform (yellow), smooth (red), pustular (pink), and non-lithifying mats (black) within the Spaven Province. The numbers 1, 2, 3 reflect transects associated with the collection of the mat types. **(B)** Cartoon depicting the position within the pool of the distinct microbial structures targeted in this study. Bar = 10 km.

Of the many extant examples of living, accreting microbialites, the largest, most extensive marine stromatolite-forming ecosystem is within the hypersaline embayment of Hamelin Pool, Shark Bay, Western Australia, a UNESCO world heritage site (**Figure 1A**; Playford, 1990; Reid et al., 2003; Suosaari et al., 2016a,b). Since their discovery in the 1950’s, the stromatolites of Hamelin Pool have served as important models to understand the formation of both modern and ancient stromatolite systems (Logan et al., 1974; Playford and Cockbain, 1976; Walter, 1976; Reid et al., 2003). Based on these pioneering studies, the stromatolite-forming microbial mats of Hamelin Pool are morphologically characterized into three canonical types: (1) ‘colloform’ mats, which form structures with moderately laminated carbonate fabrics; (2) ‘smooth’ mats, which are associated with highly laminated structures; and (3) ‘pustular’ stromatolite-forming mats, which exhibit no internal lamination (Logan et al., 1974; Playford and Cockbain, 1976). Historically, all carbonate build-ups within Hamelin pool have been referred to as ‘stromatolites’ regardless of the degree of lamination (Logan et al., 1974; Awramik et al., 1976; Playford, 1990; Jahnert and Collins, 2012; Suosaari et al., 2016a).

Each of these distinctive stromatolite-forming mat types is found in different tidal zones within the pool (Playford and Cockbain, 1976; Jahnert and Collins, 2011; Suosaari et al., 2016b). The colloform stromatolite mats are the most seaward, located in the subtidal zone and can be found in depths up to four meters, whereas the pustular stromatolite-forming mats are the most shoreward in the intertidal zone (**Figure 1B**). In addition to these three prevalent stromatolite-forming mat communities, there are non-lithifying microbial mats in the upper intertidal zone including a smooth mat type and a pustular mat type (Wong et al., 2015; Suosaari et al., 2016b). These non-lithifying mats do not develop carbonate build-ups, but rather form extensive sheets, or fields of mats, along the coastline (Suosaari et al., 2016a).

The locations of the dominant stromatolite-forming mat types and morphological variations of stromatolites within Hamelin Pool have been extensively surveyed (Jahnert and Collins, 2013; Suosaari et al., 2016a,b). A recent mapping effort revealed eight geographical zones, or “Provinces,” each with distinctive and differentiated stromatolite macrostructures (**Figure 1A**; Suosaari et al., 2016a,b). One of the largest zones is Spaven Province, located along the western edge of the pool, spanning more than 20 km. The Spaven Province is distinguished by elongated and nested stromatolites, with the direction of the elongation being perpendicular from the shore (Suosaari et al., 2016b). All three of the canonical stromatolite-forming mat types, as well as the non-lithifying mats, are present within the Spaven Province; however, no in-depth metagenomic analyses have been completed for stromatolite-forming mats in this portion of Hamelin Pool.

Over the past decade, several studies have begun to characterize the microbial diversity associated with the stromatolite-forming and non-lithifying mats within the southern region of Hamelin Pool by targeting the SSU rRNA gene (Burns et al., 2004; Papineau et al., 2005; Goh et al., 2009; Wong et al., 2015, 2017; Suosaari et al., 2016a). These studies indicate that microbial populations within the different mat types are distinctive, with minimal heterogeneity (Wong et al., 2015; Suosaari et al., 2016a). More recently, metagenomic sequencing was used to compare the non-stromatolite forming smooth and pustular mats, with an intertidal, columnar stromatolite build-up from the Nilemah province (Ruvindy et al., 2016). These results indicated that the stromatolite-forming communities exhibit a metabolic potential that is distinct from that of non-lithifying smooth and pustular mats.

In this study, we build on this previous work by comparing the metagenomes of the three main lithifying stromatolite-forming mats (i.e., colloform, smooth, and pustular) to assess whether there are distinctive metagenomic signatures for each type. We also examine the non-lithifying pustular sheet-forming mat in the upper intertidal zone, as a control, to ascertain whether there are significant differences between the lithifying and non-lithifying mats within the Spaven Province. By employing a comparative metagenomic approach, the metabolic potential for each of the dominant mat types can be identified, thereby advancing our understanding of microbes and processes associated with stromatolite formation and accretion within Hamelin Pool.

## Materials and Methods

### Sample Collection

Stromatolite-forming and non-lithifying mats were collected from the Spaven Province of Hamelin Pool, Shark Bay, Western Australia in April 2014. Although the various mat types are found throughout Hamelin Pool, relative abundance at specific locations is variable. Replicate stromatolite heads collected for this study spanned approximately 10 km within the Spaven Province (**Table 1** and **Figure 1A**). Samples from each replicate head and non-lithifying sheet mats were collected using a sterile Harris 8.0 mm Uni-Core sampler (Ted Pella, Inc., Redding, CA, United States), immediately placed in RNAlater (Life Technologies, Inc., Grand Island, NY, United States), and stored at -20°C. Upon returning to the Space Life Science Lab, cores were stored at -20°C until DNA extraction.

**Table 1:** Sample collection metadata.

Mat Type Sample^a^	Quality trimmed^b^	Annotated^c^	Filtered^d^	KEGG Annotated^e^	SSU rRNA^f^
Colloform 1	25,640,027	24,440,441	5,783,177	4,278,942	83,075
Colloform 2	25,853,795	24,806,582	6,337,080	4,783,657	76,945
Colloform 3	32,157,886	30,577,393	7,762,622	5,850,001	73,383
Colloform 4	13,882,116	13,000,926	2,837,561	2,093,877	60,831
Colloform 5	16,491,656	15,816,629	3,894,429	2,911,914	51,761
Smooth 1	12,067,338	11,623,865	2,828,663	2,122,546	45,709
Smooth 2	15,005,964	14,327,705	3,867,244	2,977,815	29,858
Smooth 3	24,026,888	22,637,278	5,074,119	3,768,477	81,085
Smooth 4	37,762,998	35,922,198	8,845,909	6,690,571	104,499
Pustular 1	43,977,296	41,753,668	10,976,883	8,126,808	114,975
Pustular 2	6,650,194	6,369,761	1,503,861	1,077,965	30,704
Pustular 3	14,791,472	14,113,834	3,943,770	2,988,764	42,968
Pustular 4	26,210,855	24,847,807	7,149,138	5,279,132	66,797
Non-lithifying 1	25,326,367	23,995,391	6,602,867	4,956,857	68,162
Non-lithifying 2	25,929,115	24,674,737	6,720,601	5,008,672	76,140
Non-lithifying 3	25,964,103	24,811,097	6,584,807	4,893,546	90,516

*^a^Each mat type sample represents a different sampled head. ^b^Reads were trimmed with sickle and quality trimmed sequences retained a Phred score > 20. ^c^Quality sequences were annotated with BLASTx against the Uniprot Swiss-prot database.^d^Sequences were filtered with a BLASTx e-value cutoff of <0.001. **^e^**Number of filtered sequences with an annotation to a KEGG Orthology (KO) level 4 gene.^f^SSU rRNA sequences were mined with SortMeRNA against the SILVA database*.

### DNA Extraction and Sequencing

Total genomic DNA was isolated (*n* = 8–10 extractions) from each replicate mat type samples using a xanthogenate bead beating method as previously described (Foster et al., 2009; Khodadad and Foster, 2012). Several modifications were made due to the high exopolysaccharide content of the mats. Each extraction replicate (60 mg) underwent three freeze-thaw cycles in liquid nitrogen, followed by immediate vortexing at max speed for 5 min with zirconia beads (Biospec Products, 2.0, 0.7, 0.1 mm diameter). All samples were visually inspected after the freeze-thaw incubations to ensure sufficient cell lysis. For those samples that did not exhibit full cell lysis due to extensive carbonate precipitate within the stromatolite-forming mat, samples were ground in liquid nitrogen prior to DNA extraction. Following extraction, DNA precipitation was conducted with 100% cold ethanol at -80°C, centrifuged, re-suspended in 70% cold ethanol, centrifuged and air dried for 2–5 min. DNA recovery was achieved with the DNeasy PowerSoil Kit (Qiagen, Germantown, MD, United States). Lastly, a second round of DNA precipitation was conducted with a final 0.3 M sodium acetate solution with cold 100% ethanol at -80°C to ensure removal of exopolysaccharides. DNA concentration was measured with Qubit^®^ 2.0 fluorometer (Thermo Fisher Scientific, Waltham, MA, United States), normalized, and replicate extractions (*n* = 8 – 10) were pooled. DNA was sequenced at the University of Florida’s Interdisciplinary Center for Biotechnology Research using the NextSeq 500/550 High Output v2 Kit (paired-end, 150 cycles) on a NextSeq 500 sequencing system (Illumina, San Diego, CA, United States). All raw reads have been deposited in the NCBI Sequence Read Archive under BioProject number PRJNA429237.

### Taxonomic Classification and Functional Annotation of Metagenomes

Raw sequences were quality filtered using sickle v1.33 with default parameters (e.g., minimum values of 20 bp length and Phred score of >20) for paired-end reads (Joshi and Fass, 2011). The range of alignment lengths for all 16 metagenomes ranged from 9 to 106 bp with a median of 48 bp. Additionally, the bit scores ranged from 20 to 125 with a median of 58 for all 16 metagenomes. For taxonomic classification, SSU rRNA sequences were recovered with SortMeRNA v2.1 using default parameters (Kopylova et al., 2012) against the SILVA_128 SSU Ref NR99 (Quast et al., 2013) database. Next, the SSU rRNA sequences were analyzed with QIIME version 1.9.1 (Caporaso et al., 2010) using the UCLUST method [open-reference operational taxonomic unit (OTU) picking] against the Silva^1^ and Greengenes v13.8 (DeSantis et al., 2006) databases. For further taxonomic analysis, the subsequent OTU table was inputted into MEGAN version 6.7.15, using the lowest common ancestor method with default parameters (Huson et al., 2016).

Quality controlled reads were annotated using BLASTx v2.2.26+ directly on the unassembled reads (Altschul et al., 1990) against the 2016 non-redundant UniProtKB/Swiss-Prot database (The UniProt Consortium, 2017) and filtered with an *E*-value cut-off of 10^-3^. An assembly free approach was used due to the variable taxonomic abundances within the stromatolite-forming mats, which has been shown to increase chimera formation frequency (Howe and Chain, 2015; Ghurye et al., 2016). Filtered BLAStx hits were programmatically linked to KEGG Orthology (KO) (Kanehisa et al., 2004) identifiers using the UniProt Retrieve/ID mapping tool. Unique KOs with their respective counts were then inputted into MEGAN to facilitate annotation of KEGG pathways (Huson et al., 2016). Pathway information for KOs not included in the MEGAN database, which was 62.3%, were identified via manual lookup on the KEGG website^2^.

### Visualization and Statistical Analysis

To preserve the biological variability and variances within the metagenomic library sizes, the raw OTU table (absolute count) was normalized using the DESeq2 normalization technique (Love et al., 2014) with QIIME. Taxonomic diversity analysis was performed on each of the samples from the normalized OTU counts. For alpha diversity metrics, Shannon–Weaver (Shannon and Weaver, 1949) and Faith’s Phylogenetic Diversity (Faith, 1992) were used and significance of the diversity indices was performed with an adonis test (i.e., an analog of the nonparametric permutational manova) using 999 permutations. Beta-diversity metrics were generated from the unweighted UniFrac distance matrix and significance was determined using an adonis test. Phyla abundances were compared between any two mat types using a Wilcoxon test and statistical significance was et at *p*-value < 0.05 and visualized using the R package MetacodeR (Foster et al., 2017). Differential abundance analysis of the KEGG orthologs was conducted using the DESeq2 v1.16.1 statistical package in R (Love et al., 2014), which has been shown to be highly effective in comparing metagenomic data (McMurdie and Holmes, 2014; Rodriguez-R et al., 2015; Warden et al., 2016). As input, DESeq2 requires un-normalized count data for the statistical model to hold as it is designed to account for library size differences internally. Statistically significant differences between pairs of mat types were identified using a negative binomial Wald test; raw *p*-values were corrected for using the Benjamin–Hochberg adjustment and all adjusted *p*-values < 0.05 were considered to indicate differentially abundant KEGG orthologs between the mat types. Orthologs (i.e., KEGG level 4) were then mapped to their respective pathways (i.e., KEGG level 3) and results of the differential analysis were visualized with ggplot2 (Wickham, 2009). PCoA of the DESeq2 normalized gene counts was conducted using vegan v2.4-4 (Oksanen, 2017) with Bray–Curtis distances as input and visualized with ggplot2.

## Results

### Site Description of Stromatolite-Forming Mats in Spaven Province

**FIGURE 2:**
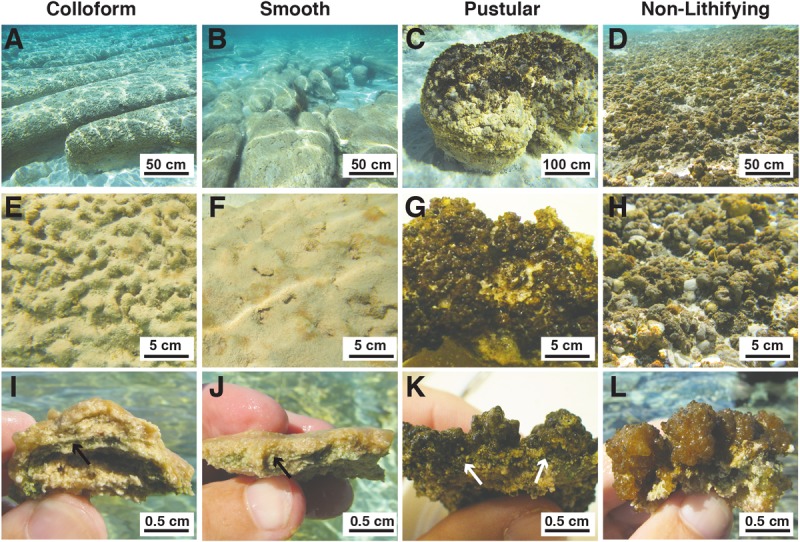
Morphological features of mat types targeted in this study. **(A–D)** Underwater images depicting the macroscopic morphologies of the elongated nested build-ups associated with the colloform **(A)** and smooth **(B)** mat types, as well as the circular carbonate build-ups associated with the pustular **(C)** stromatolite-forming mats. The non-lithifying pustular mats form extensive sheets within the upper intertidal zone. **(A,B)** Bar = 50 cm; **(C,D)** Bar = 100 cm. **(E–H)** Surface images of the mat types showing the convoluted colloform **(E)**, flat smooth **(F)**, and nodular surfaces of the lithifying **(G)** and non-lithifying **(H)** pustular mats. Bar = 5 cm. **(I–L)** Cross-section of the mat surfaces depicting the domal structure of the colloform mat **(I)**, flat surfaces of the smooth mats **(J)**, heavily pigmented nodular structure of the lithifying pustular mat **(K)**, and lighter pigmentation in the non-lithifying pustular mats **(L)**. Bar = 0.5 cm. Arrows indicate cyanobacterial layer within the stromatolite forming mats.

Within the Spaven Province, the three canonical, stromatolite-forming mat types were abundant; however, only the colloform and smooth mat types were associated with the elongated, nested stromatolite morphology characteristic of the Province (**Figures 2A,B**). Both the colloform and smooth stromatolites ranged from between 50 and 75 cm high and several meters in length. The pustular stromatolite-forming mats were associated with rounded, discrete heads that ranged from 30 to 50 cm in height and up to 1 m in width (**Figure 2C**). The non-lithifying mats formed sheets that extended for 100s of meters in the intertidal to supratidal zones (**Figure 2D**). The surfaces of each mat type showed distinctive features. The colloform stromatolites (**Figure 2E**) exhibited convoluted surfaces, whereas the smooth stromatolite-forming mats typically had flat, relatively unbroken surfaces (**Figure 2F**). Both the stromatolite-forming and non-lithifying pustular mats exhibited nodular surfaces, although the surface community on the stromatolite-forming pustular mats was darkly pigmented and often crusty compared to the non-lithifying mats (**Figures 2G,H**). In cross-section, all three of the stromatolite-forming mats exhibited extensive precipitation with a pronounced layer of cyanobacteria a few mm beneath the surface (**Figures 2I–K**), whereas the non-lithifying mat did not exhibit any lithification and often crumbled upon examination (**Figure 2L**).

Environmental data collected at the time of sampling as part of an independent study (Suosaari et al., 2016a,b) showed no significant temperature or tidal differences throughout the 20 km area of Spaven Province. The annual mean pH of the Spaven Province is 8.1 (Oehlert and Suosaari, personal communication) and the April water temperatures in Spaven Province at the collection sites ranged from 25.2 to 26°C at noon (the time of collection); salinity varied from 54.5 to 56 ppt.

### Overview of Metagenomic Sequencing of Spaven Stromatolite-Forming Mats

Replicate metagenomic libraries were generated for each of the colloform, smooth, and pustular stromatolite-forming mats (**Table 1**). Additionally, libraries were also created for non-lithifying pustular mats to enable a direct comparison with the stromatolite-forming mats. The high-throughput sequencing effort produced a total of 16 metagenomes that contained 380,819,456 raw reads with an average of 23,801,216 per sample. For downstream analysis, an average of 2.4% of the sequences were removed to produce high-quality libraries (Phred quality score > 20; **Table 1**).

For taxonomic classification, initial assessment of the data revealed that the stromatolite-forming mat communities were more than 99% bacterial with archaea and eukaryotes comprising less than 1% of the total recovered reads. Few viral sequences were recovered and viruses were likely missed due to the DNA extraction and library preparation approaches used in this study. Bioinformatic mining of the SSU rRNA sequences from the metagenomes was conducted, producing an average of 65,588 SSU rRNA sequences per sample (**Table 1**). For functional annotation, all sequences were blasted against the UniProtKB/Swiss-Prot database, rendering the deduction of functionality from manually curated protein products and filtered with an *e*-value < 0.001, generating an average of 4,238,097 reads/sample (**Table 1**) that contained a KEGG Orthology (KO) identifier.

### Comparison of Overall Microbial Diversity by Mat Type and Location

**FIGURE 3:**
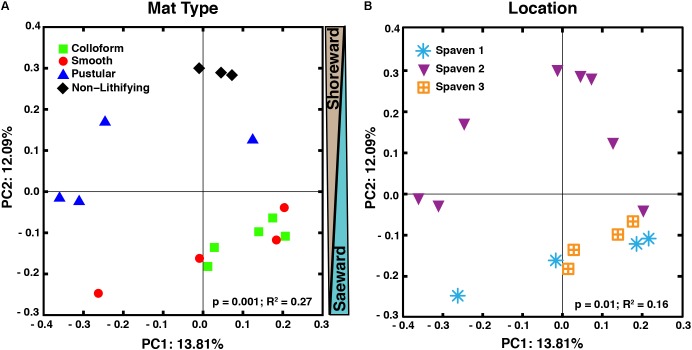
Comparison of microbial diversity between lithifying stromatolite-forming mats and non-lithifying microbial mats in Spaven Province. Principal coordinates analysis plotting the unweighted Unifrac distances between samples according to mat type **(A)** and the three collection transects (Spaven 1, 2, 3) within Spaven Province **(B)**.

The taxa of the replicate metagenomes were compared using a principal coordinates analysis (PCoA) computed from the unweighted UniFrac distances between samples (**Figure 3**). When all OTUs (i.e., bacteria, archaea, eukaryotes) associated with the metagenomes were analyzed, three groupings were discernable (**Figure 3A**; adonis, *p* = 0.001; *R*^2^ = 0.27). First, the non-lithifying mats formed a cluster that was distinctive from the three stromatolite-forming mats. Second, the pustular stromatolite-forming mats formed a separate, although less cohesive, grouping, suggesting there is a higher level of heterogeneity within that mat type. Lastly, the colloform and smooth stromatolite-forming mats formed a single grouping, indicating a high degree of similarity between these two mat types. As sample collection spanned several km, the taxa of the metagenomes were also compared based on location within Spaven Province; **Figure 3B**). The taxonomic differences between the mat types was weakly linked to the collection area (adonis, *p* = 0.01; *R*^2^ = 0.16) and clustered primarily based on mat type.

### Taxonomic Assessment of the Spaven Stromatolite-Forming Mats

Analysis of the 16 metagenomes revealed 33 bacterial, 3 archaeal and 3 eukaryotic phyla or superphyla. The overall alpha diversity within the different mat types was examined using both Shannon-Wiener Diversity and Faith’s Phylogenetic Diversity metrics (**Supplementary Figures S1A,B**). No statistical differences for either metric were observed (*p* > 0.05), indicating that the overall level of microbial diversity between the different mat types was comparable. The indices values were, however, much higher than previous reports (Allen et al., 2009; Goh et al., 2009; Garby et al., 2013; Wong et al., 2015; Suosaari et al., 2016b) likely reflecting the use of newer sequencing platforms (e.g., Illumina) that lead to an increase in the overall microbial diversity detected within all mat types compared to previous technologies.

**FIGURE 4:**
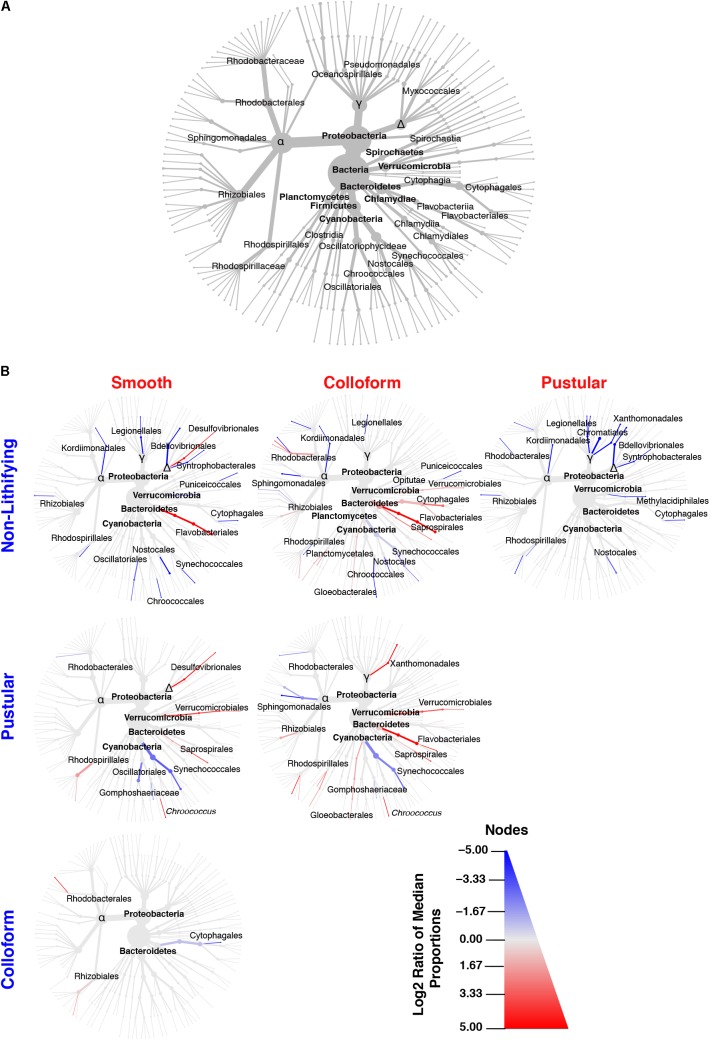
Taxonomic distribution of bacteria within the targeted mat types. **(A)** Overview of the top 35 bacterial taxa recovered from the 16 metagenomic libraries generated in this study and serves as orientation for **(B)**. **(B)** Differential heat trees of pairwise comparisons between different mat types highlighting significant taxonomic enrichments (Wilcoxon test, *p* < 0.05). The color of each taxon represents the log-2 ratio of the median of the proportions observed with each mat type and corresponds to the different pairwise comparisons listed along the columns or rows. Node/edge color and size display the relative proportion for each taxon with lines in red reflecting those taxa enriched in mats along the *x*-axis and lines in blue enriched along the *y*-axis.

In all the different mat types, the dominant bacterial phyla included the Proteobacteria (55–69%) and Cyanobacteria (15–29%), with the Planctomycetes (5–7%), Bacteroidetes (3–7%), Verrucomicrobia (2–3%), Chloroflexi (2–5%), and Actinobacteria (0.25–2%) phyla present in all of the mat types (**Figure 4A** and **Supplementary Figure S1C**). At much lower abundances (relative abundance < 1% across all replicates), a total of 26 additional bacterial phyla were observed (**Supplementary Figure S1D**). Although bacteria dominated all mat types, the recovered archaea were predominantly Thaumarchaeota (**Supplementary Figure S1E**) whereas the eukaryotic taxa were primarily associated with the Opisthokonta and the SAR supergroup, which includes the stramenopiles, alveolates and Rhizarias (**Supplementary Figure S1F**). These results are consistent with previously documented taxa within the southern region of Hamelin Pool (Burns et al., 2004; Goh et al., 2009; Edgcomb et al., 2014; Wong et al., 2015; Ruvindy et al., 2016; Suosaari et al., 2016a).

Although few differences were observed between mat types at the phylum-level, in-depth pairwise comparisons of the bacteria revealed significant differences at lower taxonomic levels (Wilcoxon test, *p* < 0.05) and are visualized as differential abundance heat trees in **Figure 4B**. The most pronounced differences occurred between the stromatolite-forming mat types and the non-lithifying mats. There was a pronounced overall enrichment of Proteobacteria, in particular, Alpha-, Delta- and Gammaproteobacteria taxa in the non-lithifying mats compared to the stromatolite-forming mat types (**Figure 4B** and **Supplementary Figure S2**); however, some individual proteobacterial taxa were enriched in the stromatolite-forming mats. For example, in the smooth mats, there was a statistically significant enrichment of Desulfovibrionales compared to the non-lithifying mats, whereas in the colloform mats increases in several Rhodobacterales taxa were observed. In the colloform and smooth stromatolite-forming mats, there was also an enrichment of Bacteroidetes, specifically in the Flavobacteriales. The colloform mats also were enriched in Saprospirales and Cytophagales compared to the non-lithifying mats.

Differences were also observed between the three dominant stromatolite-forming mat types. The most pronounced difference was an enrichment of the coccoid cyanobacteria Synechococcales within the pustular mats compared to the colloform and smooth mat types (**Figure 4B** and **Supplementary Figure S3**; Wilcoxon test, *p* < 0.05). There was also a differential abundance of two different Chroococcales lineages. The colloform and smooth mats were enriched in the genus *Chroococcus*, whereas the pustular mats were enriched in the Gomphosphaeriaceae family. Both the smooth and colloform mats showed an enrichment of Verrucomicrobia and Bacteroidetes compared to the pustular stromatolite-forming mats. There were very few differences observed between the colloform and smooth stromatolite-forming mats with an increased abundance of the Cytophagales in the colloform mats and a few enriched Alphaproteobacteria (Rhodobacterales and Rhizobiales) in the smooth stromatolite-forming mats (**Figure 4B**).

### Comparison of the Functional Genes Within the Stromatolite-Forming Mats

**FIGURE 5:**
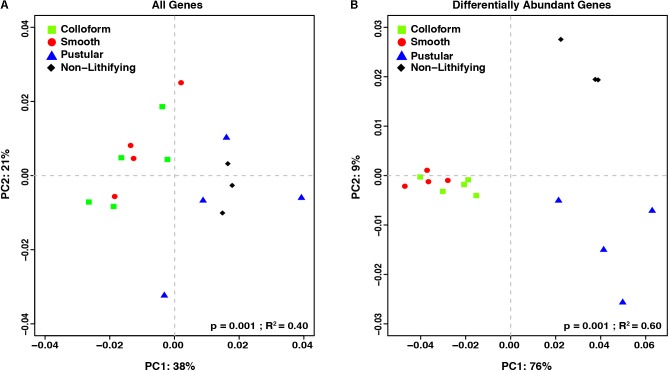
Comparison of the functional gene profiles between the stromatolite-forming and non-lithifying mat types. Principal coordinate analysis (PCoA) of all DESeq2 normalized KEGG level 4 gene counts of **(A)** all recovered genes and **(B)** differentially abundant genes (*p*-value < 0.001) from the different mat types.

To complement the taxonomic comparison, an ortholog-based comparison between the different mat types was performed using PCoA analysis of KEGG orthologies (**Figure 5**). When all annotated genes were considered, there were primarily two distinct clusters, one that included the colloform and smooth stromatolite-forming mats and a second that contained the pustular stromatolite-forming mats and non-lithifying mats (**Figure 5A**; adonis, *p* = 0.001; *R*^2^ = 0.40).

In an effort to further characterize the metabolic differences between the mat types, pairwise comparisons of the differentially abundant genes were conducted with DESeq2 (**Table 2**; Love et al., 2014). There were 1232 differentially abundant genes (*p* < 0.05) between the non-lithifying mats and the colloform stromatolite-forming mats and 1033 between the non-lithifying mats and smooth stromatolite-forming mats, respectively (**Table 2**). A PCoA visualization was performed with statistically significant genes (*p* < 0.001), as was determined by differential abundance analysis with DESeq2 (**Figure 5B**). This approach more clearly revealed differences between the non-lithifying and pustular mat types, however, the colloform and smooth mats were still tightly clustered.

**Table 2:** Differential gene abundance comparison between lithifying and non-lithifying mat types abundance mat types.

Pairwise Comparison	Genes^a^	*p* < 0.05	Genes *p* < 0.001	Unique^b^ pathways (p < 0.001)
Colloform vs. Smooth	4	–	–
Pustular vs. Smooth	1,070	394	108
Pustular vs. Colloform	1,248	318	98
Non-Lithifying vs. Colloform	1,232	261	105
Non-Lithifying vs. Smooth	1,033	231	92
Non-Lithifying vs. Pustular	210	36	28

*^a^KEGG Level 4 genes. ^b^Unique KEGG Level 3 pathways*.

Interestingly, there were only four significant differentially abundant genes (*p* < 0.05) between the colloform and the smooth stromatolite-forming mat types, suggesting the genetic profiles of the microbes in these communities are highly similar. For those pairwise comparisons that had >1000 differentially abundant genes, filtering criteria were employed to identify those genes with a *p*-value < 0.001 and an absolute log2-fold change > 1 (**Table 2** and **Supplementary Table S1**).

In pairwise comparisons between the shoreward, non-lithifying mats and the stromatolite-forming mats, there were enrichments in numerous glycan and polysaccharide biosynthesis and degradation genes in the colloform and smooth stromatolite-forming mats, such as beta-porphyranases, fuctosidases, fructosideases, and rhamnotransferases (**Supplementary Figures S4–S7**). These genes were associated with a wide distribution of taxa including Chroococcales, Planctomycetales, several orders of Bacteroidetes, Firmicutes, and Proteobacteria (**Supplementary Figures S8–S12**). There was also a prevalence of genes associated with dissimilatory sulfur metabolism in the colloform and smooth stromatolite-forming mats compared to the non-lithifying mats, such as genes encoding thiosulfate reductases, dimethyl sulfoxide reductases (**Supplementary Figures S4–S7** and **Supplementary Table S1**). There were fewer significant differences between the non-lithifying and pustular stromatolite-forming mats with the highest differentially abundant gene with regard to fold change associated with pigment transport in the pustular stromatolite and several uncharacterized proteins (**Supplementary Figures S4–S7** and **Supplementary Table S1**).

In the upper intertidal, non-lithifying mats there was a higher representation of genes associated with oxidative and osmotic stress responses, including nitric oxide reductases, peroxidases, glucosylglycerol-phosphate synthase (GgpP), and glycine betaine/proline transporters (**Supplementary Figures S4–S7**). These genes were associated with a wide range of Proteobacteria, including several methylotrophic bacteria and purple-sulfur bacteria (**Supplementary Figures S8–S12**). There was also a significant enrichment of genes associated with metalloid and heavy metal cycling in the non-lithifying mats, such as arsenite transporters (e.g., *arsAB*), arsenite-mycothiol transferase (arsC) for removal, arsenic resistance genes (e.g., *arsH*), and arsenite oxidases (e.g., *aoxAB*) as well as genes that are part of the cobalt-zinc-cadmium efflux system (**Supplementary Figures S4–S7**). Genes associated with arsenate reductase were recovered from the non-lithifying mats, but they were not differentially abundant compared to the stromatolite-forming mat types. The genes associated with arsenic metabolism were widespread in the Actinobacteria, Cyanobacteria, and Firmicutes phyla, whereas the genes associated with the cobalt-zinc-cadmium system were found only in the Firmicutes and cyanobacterial Synechococcales (**Supplementary Figures S8–S12**).

**FIGURE 6:**
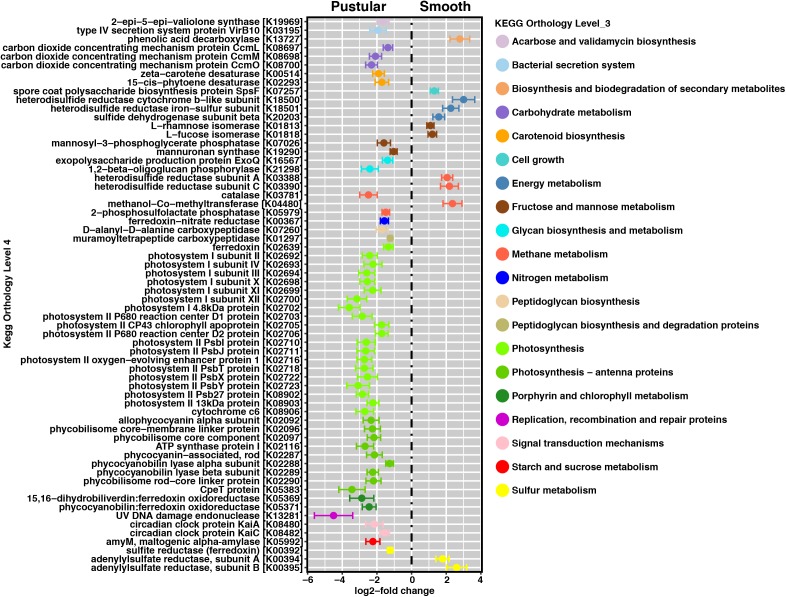
Pairwise comparison of differentially abundant genes between pustular and smooth stromatolite-forming mats. Representative differentially abundant genes (*p* < 0.001) colored by their respective pathways. A positive log2-fold change designates differential abundance in the smooth mat type and a negative log2-fold change designates differential abundance within the pustular mats. Lines indicate the standard error of the mean.

**FIGURE 7:**
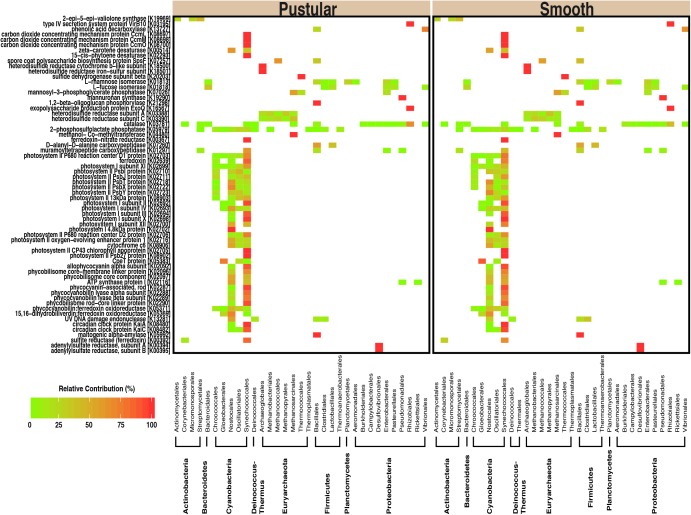
Taxa associated with selected functional pathways. Heatmap showing the relative contribution of each taxon with respect to the differentially abundant genes within the pustular and the smooth stromatolite-forming mats.

In pairwise comparisons between the different stromatolite-forming mats, the most significant differentially abundant genes were observed in the pustular stromatolite-forming mats (**Figure 6** and **Supplementary Figures S4–S7**). There was an increase in the abundance of genes associated with carbon-concentrating mechanisms and photosynthesis compared to the colloform and smooth stromatolite-forming mats (*p* < 0.001; **Figure 6** and **Supplementary Figures S4–S7**). These differentially abundant photosynthesis genes were associated with both photosystem I and II pathways, as well as numerous antenna proteins that included phycocyanins and the blue phycocyanobilin (**Supplementary Table S1**). The differentially abundant photosynthesis genes were widely distributed in the coccoid Chroococcales and Synechococcales orders as well as the filamentous Nostocales cyanobacteria (**Figure 7** and **Supplementary Figures S8–S12**). As with the non-lithifying mats, the colloform and smooth stromatolite-forming mats were enriched in genes associated with heterotrophic metabolisms, such as methane and sulfur reduction metabolisms. Differentially abundant genes associated with these pathways included co-methyltransferase, heterodisulfide reductase, thioredoxin reductase, adenylylsulfate (APS) reductases and sulfide dehydrogenases (**Supplementary Table S1**). The methanogenesis genes were associated with Euryarchaeota (e.g., Methanobacteriales, Methanococcales, Methanopyrales, and Methanosarcinales), whereas the dissimilatory sulfur reduction genes were derived from the Desulfovibrionales (**Supplementary Figures S8–S12**).

## Discussion

Hamelin Pool represents the largest known marine habitat for actively accreting stromatolites; however, little is known about the molecular pathways underlying the different stromatolite-forming mat types in this ecosystem. In this study, the metagenomes of the three canonical microbial mat types associated with carbonate build-ups were compared to each other and to an adjacent sheet-forming mat to assess differences in the metabolic signatures of lithifying and non-lithifying microbial mats. The results of this study provide evidence that: (1) the stromatolite-forming mats types have distinct taxonomic and functional gene profiles compared to non-lithifying mats; (2) colloform and smooth stromatolite-forming mats exhibit distinctive surface morphologies, yet show few taxonomic and functional gene differences; and (3) intertidal pustular stromatolite-forming mats are enriched in photosynthetic genes compared to the colloform and smooth stromatolite-forming mats, suggesting two distinctive metabolic processes driving stromatolite formation within Hamelin Pool.

The metagenomic comparisons in this study provided an important, in-depth analysis of those differentially abundant metabolic pathways between lithifying and non-lithifying mat communities. Our current study expands upon previous metagenomic analyses (Ruvindy et al., 2016) to examine all three of the prevalent lithifying mat types. Our results revealed that one of the pronounced differences within the non-lithifying mats compared to the subtidal stromatolite-forming mat types was the enrichment of genes associated with metalloid cycling (*p* < 0.001), in particular, genes associated with arsenic metabolism. The presence of arsenic metabolism has been previously reported in the non-lithifying mats of the southern Nilemah Province within Hamelin Pool (Ruvindy et al., 2016), hypersaline hot spring biofilms from Mono Lake (Kulp et al., 2008), as well as within the high altitude stromatolites of Socompa Lake, Argentina (Kurth et al., 2017). The increase in abundance of these genes associated with metal cycling within the mats of the upper zone may reflect the hypersaline nature of these habitats (Hamelin Pool. 66 – 88 ppt; Mono Lake 84 ppt; Socampa Lake ∼90 ppt; Farías et al., 2013). For example, evaporation can increase the abundance and availability of metalloids, such as arsenic and boron, within microbial communities (Kulp et al., 2007). Additionally, under extreme saline conditions some metabolisms, such as sulfate reduction and methanogenesis, are diminished (Oren, 1999; Kulp et al., 2007; Wong et al., 2017) and reliance on arsenic cycling, particularly under anoxic conditions, may reflect an important metabolic strategy for these mat communities exposed to the energetically taxing high salt and desiccation conditions of the upper, intertidal zone.

Another pronounced difference within the non-lithifying mats compared to the stromatolite-forming mat types in Spaven Province was the enrichment of oxidative (e.g., nitric oxide reductases, peroxidases) and osmotic stress (e.g., glycine betaine/proline synthesis and transport) genes in the upper tidal non-lithifying mats, whereas genes typically associated with UV stress were found to be evenly represented in all of the different mat types examined. The enriched oxidative and osmotic stress genes in the non-lithifying mats corresponded to several of observed osmoadaptive genes and pathways recovered from hypersaline mat environments, including Hamelin Pool (Goh et al., 2010; Goh et al., 2011; Gudhka et al., 2015; Gunde-Cimerman et al., 2018). As there is a pronounced desiccation gradient within the pool (Burne and Johnson, 2012; Suosaari et al., 2016b), the enrichment of genes associated with the production of osmoregulation and oxidative stress responses in the non-lithifying mats likely reflects the dynamic tidal extremes within the pool. Additionally, several taxonomic analyses of the non-lithifying mats in the Nilemah Province of Hamelin Pool have shown distinctive gradations of key functional groups of microbes, such as sulfate-reducing bacteria and methanogens, at different tidal regimes further suggesting that water levels are influencing the mat communities (Wong et al., 2017). The enriched genes associated with pigment transport were associated with cyanobacteria and may reflect the higher light and salt selection pressures on the cyanobacteria within this mat type, as several cyanobacteria have shown differential pigment production and transport responses to light and salt stress (Riethman et al., 1988; Sudhir and Murthy, 2004). Several comparative genomic studies of marine cyanobacteria have revealed genome expansion as the result of nutrient or light stresses (Swingley et al., 2008) and light pressures can be a major driver of genome differences in pigments and the photosynthesis apparatus between closely related cyanobacterial ecotypes (Rocap et al., 2003).

Over the course of a year, the water levels within the pool can vary by as much as two meters and are highly dependent on seasonal, meteorological and astronomical effects (Burne and Johnson, 2012; Playford et al., 2013; Suosaari et al., 2016b). Although water levels are typically higher during the austral summer and fall, which coincided with the April collection of the samples used in this study, the location of Spaven Province on the eastern margin of the pool experiences the largest meteorological effect of tidal variation throughout the year, spanning 1.67 m (Suosaari et al., 2016a), indicating the non-lithifying mats would experience frequent exposure and desiccation throughout the diel cycle. Temperatures within the pool can also fluctuate between 11 and 33°C through the year (Suosaari et al., 2016a); however, due to the shallowness of the pool, there is no vertical temperature stratification within the water column (Burling et al., 2003). Therefore, it is more likely that water depth, has a stronger influence than temperature on driving the differences in the metagenomes between the mat types.

The concept that water depth influences the morphology and community composition of the stromatolite-forming mats has long been suggested (e.g., Logan, 1961; Logan et al., 1974; Golubic and Hofmann, 1976; Playford, 1990; Jahnert and Collins, 2011, 2012; Playford et al., 2013; Suosaari et al., 2016a; Wong et al., 2017). More recent studies suggest that water level and the underlying shelf physiography may be two of the most important drivers of microbial mat zonation within Hamelin Pool (Suosaari et al., 2016b). The PCoA comparisons of both the taxonomic and functional gene diversity (**Figures 3, 5**) of the different mat types provide support for these concepts, as the stromatolite-forming mats formed three distinct clusters that corresponded with the upper tidal, intertidal and subtidal zones.

Interestingly, within the subtidal zone, the colloform and the smooth stromatolite-forming mats showed few taxonomic and functional gene differences. In fact, only four of the 15,378 recovered genes were significantly different between colloform and smooth stromatolites (**Table 2** and **Supplementary Figure S5**) indicating that these two subtidal mat types share almost identical metabolic potential. Despite the similar metabolic profiles between the two subtidal stromatolite-forming mat types, each of the associated carbonate build-ups exhibited different surface morphologies, with the colloform mats exhibiting a convoluted surface and the smooth mats having a flat, even surface. These differences could reflect differential gene expression between the two mats, as other metatranscriptomic analyses of Bahamian thrombolites have shown that the metatranscriptome can vary significantly throughout diel and seasonal cycles and does not fully reflect the metagenome (Mobberley et al., 2015; Louyakis et al., 2018).

Alternatively, the differences in morphology may reflect environmental factors impacting the mat surfaces, specifically, the shear forces of winds and currents. Although water circulation within Hamelin Pool is restricted due to the presence of the Faure Sill in the northern part of the pool, it is primarily driven by the wind resulting in Langmuir circulation, which are shallow, counter-rotating gyres that align with the winds (Playford et al., 2013; Suosaari et al., 2016b). In the Spaven Province, current velocities range from as low as 0.001 m/s to a maximum of 0.54 m/s with a mean of 0.125 m/s (Suosaari et al., 2016b). Waves driven by the wind could be differentially impacting the colloform and smooth stromatolite-forming mat communities in the Spaven Province resulting in the disparate morphologies. Additional analyses, such as metatranscriptomics and proteomics, will be required to ascertain whether the colloform and smooth microbial communities exhibit differential microbial activities that account for the differences in morphology or whether these differences are the product of extrinsic factors.

One key difference amongst the stromatolite-forming mat communities was the enrichment of genes associated with photosynthesis in the pustular mats, including genes associated with photosystems I/II, as well as a range of pigments and antennae proteins. These differentially abundant genes were widely distributed within the cyanobacteria and were associated with the coccoid Chroococcales and Synechococcales, as well as the filamentous Nostocales orders. Due to the paucity of sequenced genomes for cyanobacteria, many of the genes were unable to be identified beyond the order level. However, over the past few decades, microscopic analyses of the pustular mats have shown that *Entophysalis* spp., which belongs to the order Chroococcales as well as the filamentous Nostocales *Scytonema* spp. and *Dichothrix* spp., dominate the pustular mat types (Logan et al., 1974; Golubic and Hofmann, 1976; Collins and Jahnert, 2014; Suosaari et al., 2016a). These taxa have long been thought to play a key role in the formation of stromatolites, both in living and in ancient systems (Golubic and Hofmann, 1976; Golubic and Campbell, 1981). Additionally, recent metagenomic and metatranscriptomic examination of the unlaminated thrombolites of Highborne Cay, The Bahamas have shown that the filamentous *Dichothrix* spp. and its associated coccoid cyanobacteria are the most transcriptionally active taxa within the thrombolite communities, with most transcripts associated with photosynthesis (Mobberley et al., 2015; Louyakis et al., 2017, 2018). These results coupled with stable isotope analyses of the calcium carbonate have revealed that the primary metabolism driving precipitation in the intertidal Bahamian thrombolites is photosynthesis (Louyakis et al., 2017).

The enrichment of photosynthetic genes in the pustular stromatolite-forming mats of Hamelin Pool suggests that, like the Bahamian thrombolites, photosynthesis may be the primary metabolism driving precipitation in this mat type. Additionally, as the carbonate build-ups associated within the pustular mats are typically unlaminated, and have been called stromatolites rather than thrombolites for historical reasons (Logan, 1961; Logan et al., 1974; Golubic and Hofmann, 1976; Playford, 1990; Jahnert and Collins, 2012, 2013; Playford et al., 2013), the processes associated with pustular carbonate build-ups may be distinct from the other lithifying mat structures and may be more thrombolite-like. Additional research into the biogeochemical and transcriptional activities of the filamentous *Dichothrix* spp. and coccoid *Entophysalis* spp. is needed to fully elucidate the role of these abundant taxa in the formation of the pustular-associated carbonate structures.

Whereas the pustular stromatolite-forming mats harbored an increase in photosynthesis genes, the colloform and smooth stromatolite-forming mats exhibited a differential abundance of genes associated with certain heterotrophic pathways known to promote carbonate precipitation. For example, there was a differential abundance of genes associated with dissimilatory sulfate reduction and methanogenesis in the colloform and smooth mats. Previous research on the stromatolites of Highborne Cay, The Bahamas has shown a strong correlation between sulfate-reducing activity and lithified micritic lamination (Visscher et al., 1998, 2000). Bicarbonate is a product of sulfate reduction, which can alter the local pH within the mat microenvironment, thereby promoting carbonate precipitation (Dupraz and Visscher, 2005; Visscher and Stolz, 2005; ?). Methanogenesis can also increase the local pH subsequently facilitating conditions for carbonate precipitation (Visscher and Stolz, 2005) and several genes associated with that pathway were enriched in the lithifying colloform and smooth mats compared to the pustular stromatolite-forming mats.

In addition to creating localized geochemical environments that favor carbonate precipitation (?), nucleation sites must also be available. The primary nucleation sites of the lithifying mats are within the exopolymeric substances (EPS) secreted outside the cells forming a matrix (Braissant et al., 2007; ?). The EPS matrix provides a multi-faceted role for the mat community, not only acting as nucleation sites for carbonate precipitation but also serves as a protective barrier against environmental stressors and enhances community stability in high-energy environments (Decho et al., 2005; Decho and Gutierrez, 2017). Within the EPS, negatively charged acidic groups can bind Ca^2+^, thereby sequestering it within the matrix (Kawaguchi and Decho, 2002) and through modification to the EPS, either by environmental or metabolic degradation, the ions can be released, thus increasing local calcium concentration available for mineralization (Visscher et al., 2000; Dupraz and Visscher, 2005; ?).

In the colloform and smooth stromatolite-forming mats, there was an increase in the abundance of genes associated with formation and degradation of EPS. Specifically, there was an increase in the abundance of metabolic enzymes associated with modification of dicarboxylic acids (e.g., maleic acid) and deoxyhexoses (e.g., fucose and rhamnose) from a wide range of taxa including Bacteroidetes, Firmicutes, Planctomycetes, and Proteobacteria compared to the pustular stromatolite-forming mats. This enrichment suggests that the EPS material of the colloform and smooth stromatolite-forming mats is highly labile and may fuel heterotrophic activity within the mats. The processing and restructuring of the EPS by heterotrophic activity may change the physiochemical properties of the EPS matrix, thereby influencing the morphology and mineralogy of the precipitates within colloform and smooth stromatolites. Together, these results suggest the processes facilitating carbonate precipitation in the laminated colloform and smooth stromatolite-forming mats may rely more on heterotrophic processes than in the unlaminated pustular stromatolite-forming mat type.

In summary, our results provide the first metagenomic comparison of the three dominant stromatolite-forming mat types within Hamelin Pool. Our results reveal key metabolic differences amongst the different stromatolite-forming mat communities, in particular between the intertidal and subtidal zones. The results suggest that carbonate precipitation in the unlaminated, pustular mat build-ups may be primarily photosynthesis driven, whereas, in the laminated colloform and smooth stromatolite structures, carbonate precipitation may be the product of a synergism between autotrophic and heterotrophic processes in the associated mats. More in-depth analysis of the microbial activities within each of these lithifying mat types will be required to more fully understand the metabolic drivers of carbonate precipitation within Hamelin Pool. Taken together, this comparative metagenomic analysis has provided confirmation of many of the prior geological and morphological-based studies in Hamelin Pool, thereby providing important insight into the feedbacks between microbial mat communities and their environments, which together drive stromatolite formation.

## Author Contributions

JF and PR conducted all the fieldwork. JB conducted the experiments, sequencing analysis, and wrote the first draft of the manuscript. AC, GC, CP, AL, and JF assisted in developing the bioinformatic pipeline and analysis of the data. All authors assisted in the writing and editing of the manuscript.

## Conflict of Interest Statement

The authors declare that the research was conducted in the absence of any commercial or financial relationships that could be construed as a potential conflict of interest.

## Footnotes

1 http://www.arb-silva.de/

2 http://www.genome.jp/kegg/kegg2.html
