# Supplementary material for: Comparative Metagenomics Provides Insight Into the Ecosystem Functioning of the Shark Bay Stromatolites, Western Australia
Source: Front Microbiol. 2018 Jun 25;9:1359. doi: 10.3389/fmicb.2018.01359 (PMC6027182; doi:10.3389/fmicb.2018.01359)
Supplement: Supplementary file 2 [file Image_1.pdf]

Supplemental Material:

Comparative Metagenomics of the Dominant Stromatolite-Forming Mats of Hamelin Pool, Shark Bay, Western Australia

Joany Babilonia[1], Ana Conesa[2,3], Giorgio Casaburi[1], Cecile Pereira[2,4], Artemis S. Louyakis[1], R. Pamela Reid[5], Jamie S. Foster[1*]

[1]Department of Microbiology and Cell Science, University of Florida, Space Life Science Lab, Merritt Island, FL, USA,

[2]Department of Microbiology and Cell Science, University of Florida, Gainesville, FL, USA,

[3]Genomics of Gene Expression Laboratory, Prince Felipe Research Center, Valencia, Spain

[4]EURA NOVA, Marseille, France

[5]Rosenstiel School of Marine and Atmospheric Science, University of Miami, Miami, FL, USA

*Corresponding Author:

Jamie S. Foster (jfoster@ufl.edu)

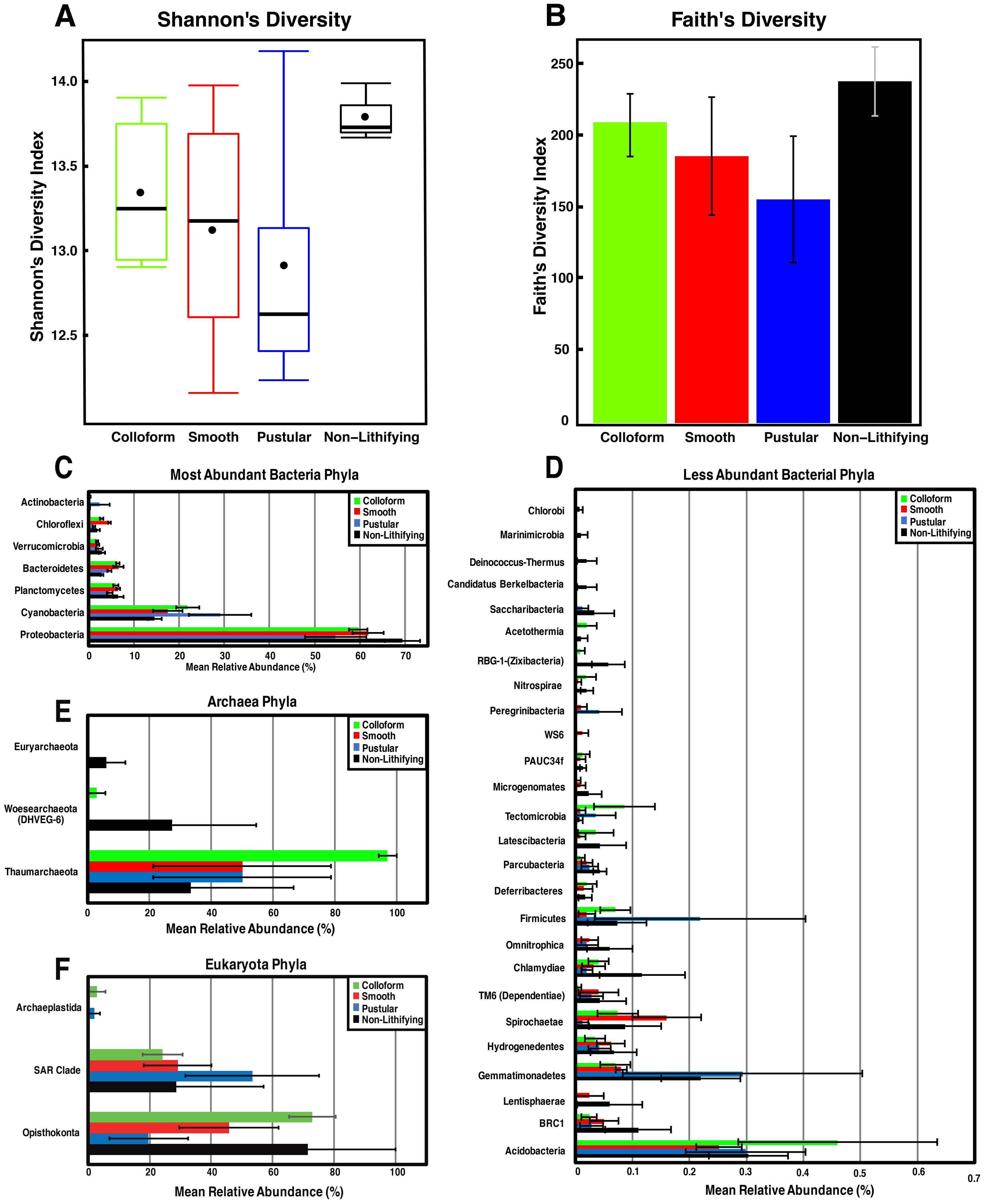

Supplemental Figure S1. Overall taxonomic diversity of dominant lithifying and non-lithifying mats within Spaven Province. Comparison of the Shannon's (A) and Faith's (B) diversity indices of each of the targeted mat types. Relative abundance of the most (C) and least (D) abundant bacterial phyla, as well as the most abundant archaeal (E) and eukaryotic (F) phyla within the different mat types. SAR clade refers to the eukaryotic supergroup that includes the stramenopiles, alveolates, and rhizarias.

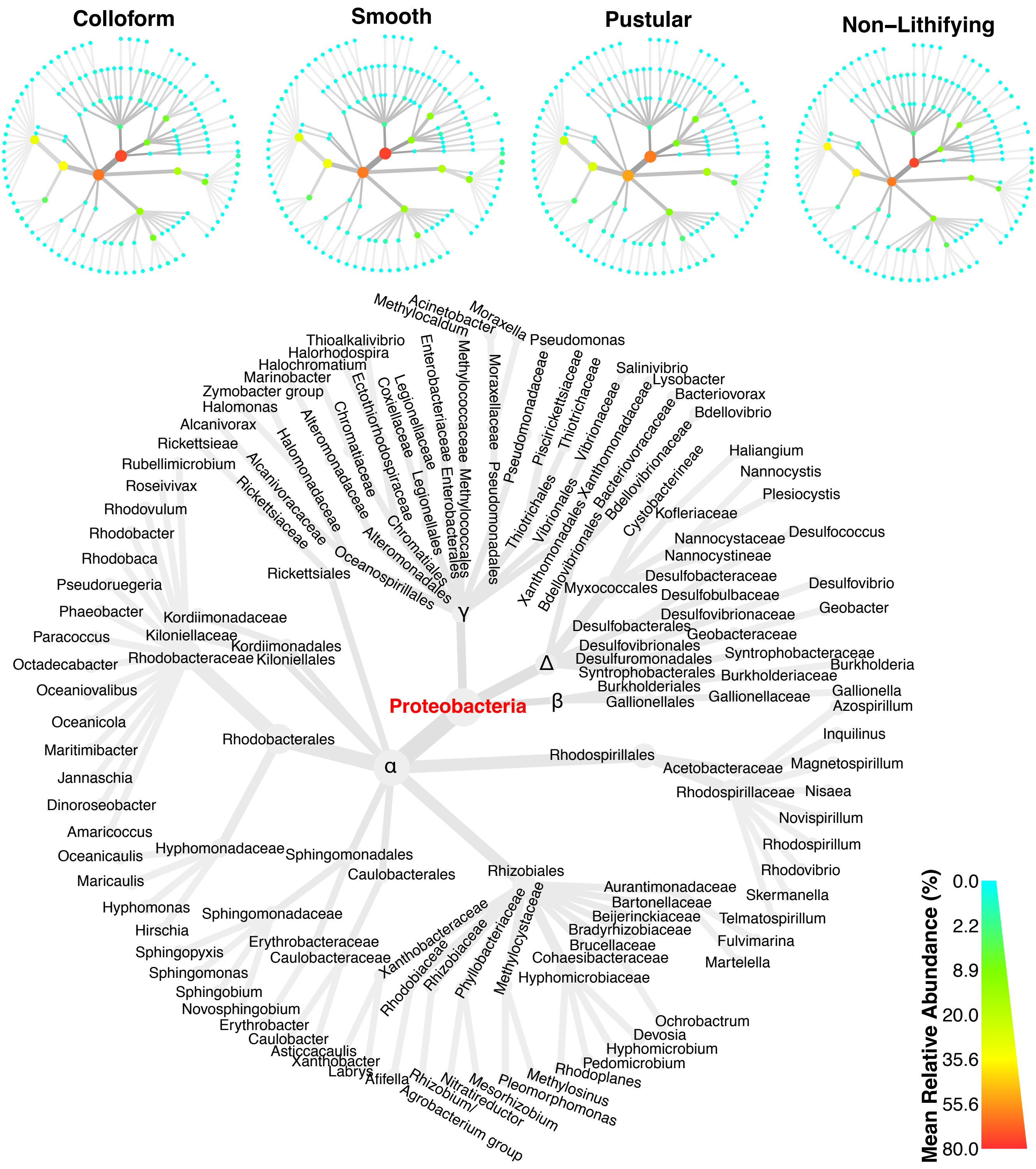

Supplemental Figure S2. Comparative heat tree for the dominant Proteobacteria within the lithifying and non-lithifying mats. Colors reflect the mean relative abundance of the different taxa. Node color and size display the relative proportion for each taxon. The larger annotated tree serves as a guide for the four smaller trees at the top of figure.

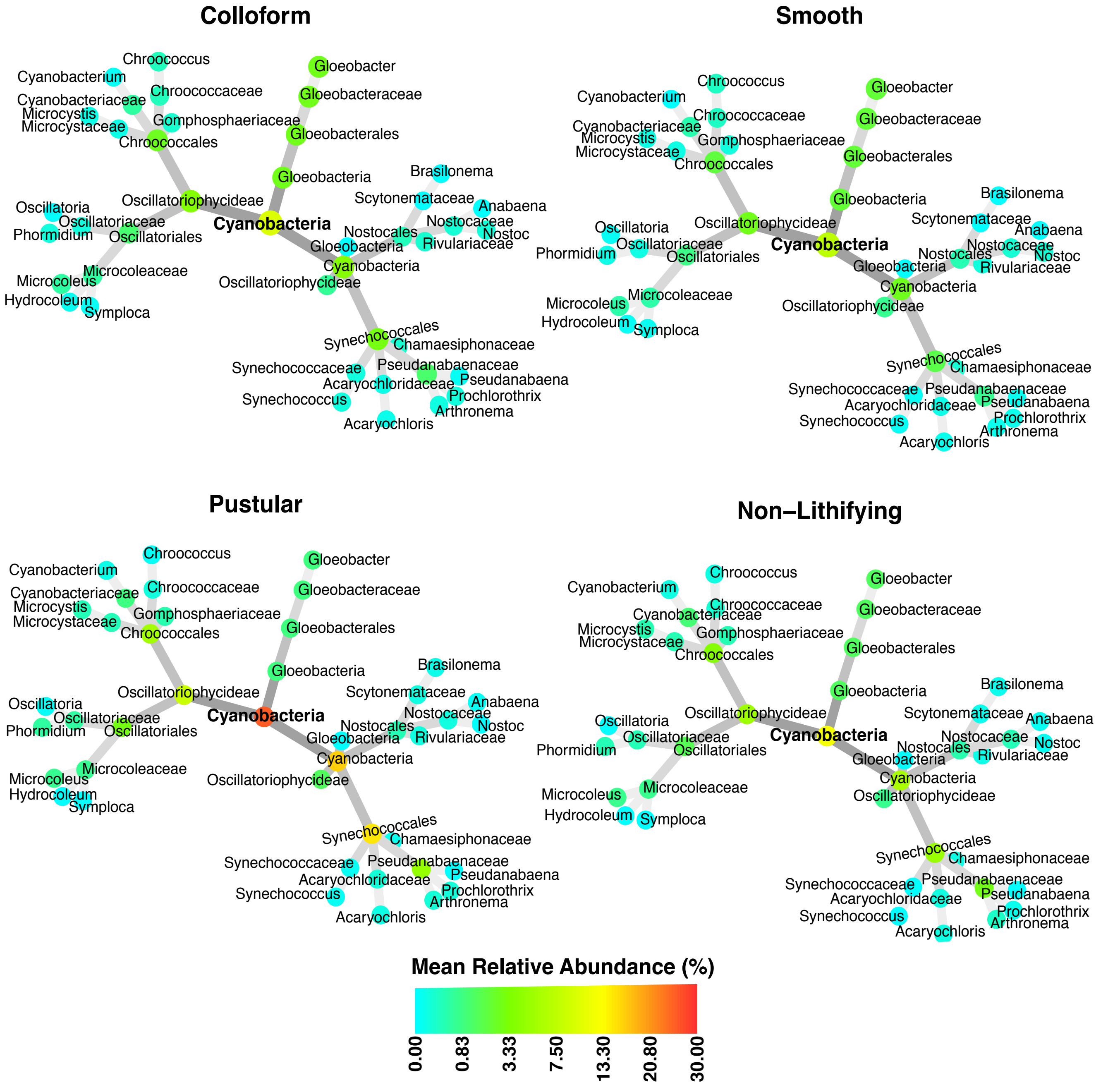

Supplemental Figure S3. Comparative heat tree for the dominant Cyanobacteria within the lithifying and non-lithifying mats. Colors reflect the mean relative abundance of the different taxa.

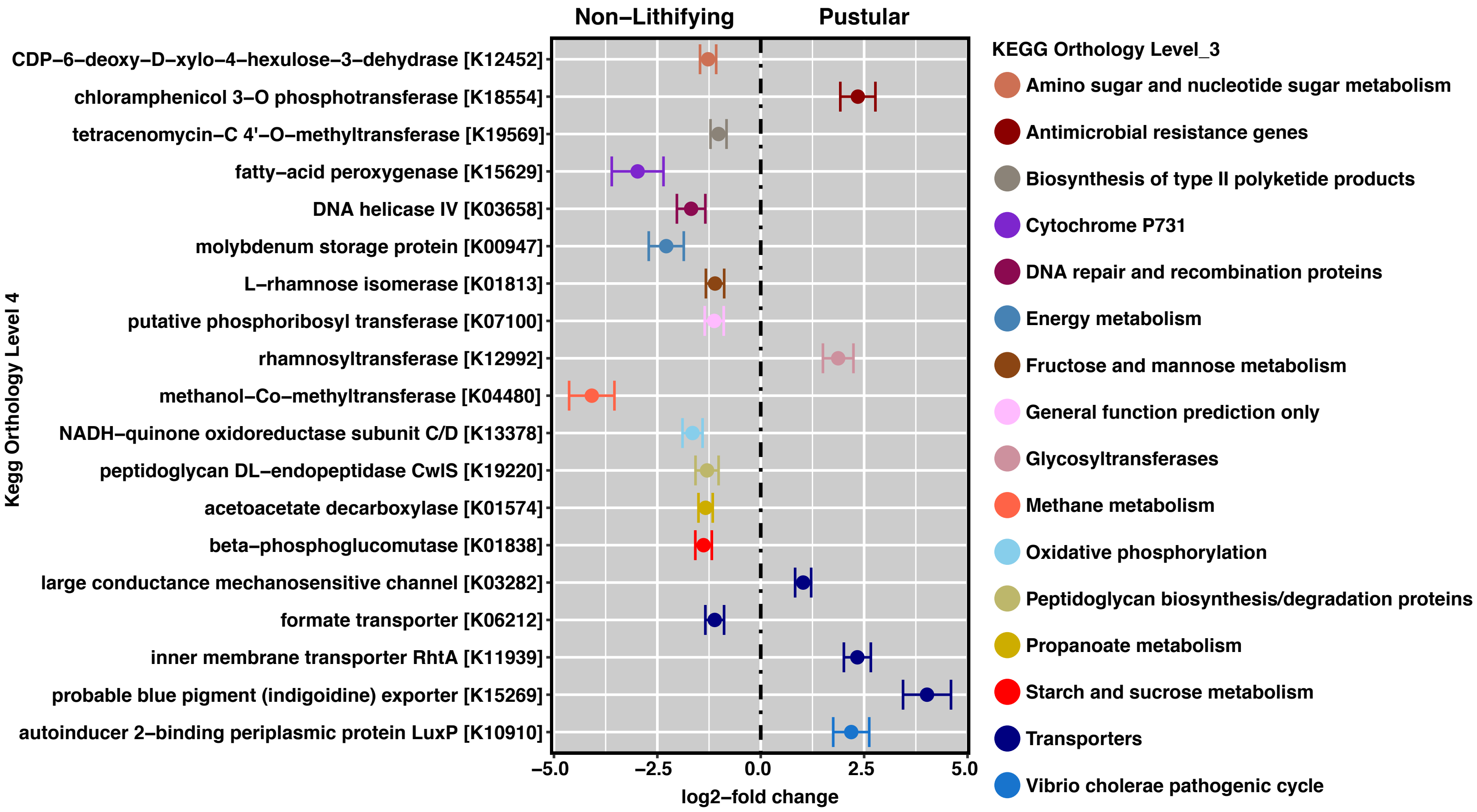

Supplemental Figure S4. Pairwise comparison of differentially abundant genes between non-lithifying and pustular stromatolite-forming mats. Representative differentially abundant genes ($p < 0.001$), colored by their respective pathways. A positive log2-fold change designates differential abundance in the pustular mat type and a negative log2-fold change designates differential abundance within the non-lithifying mats. Lines indicate the standard error of the mean.

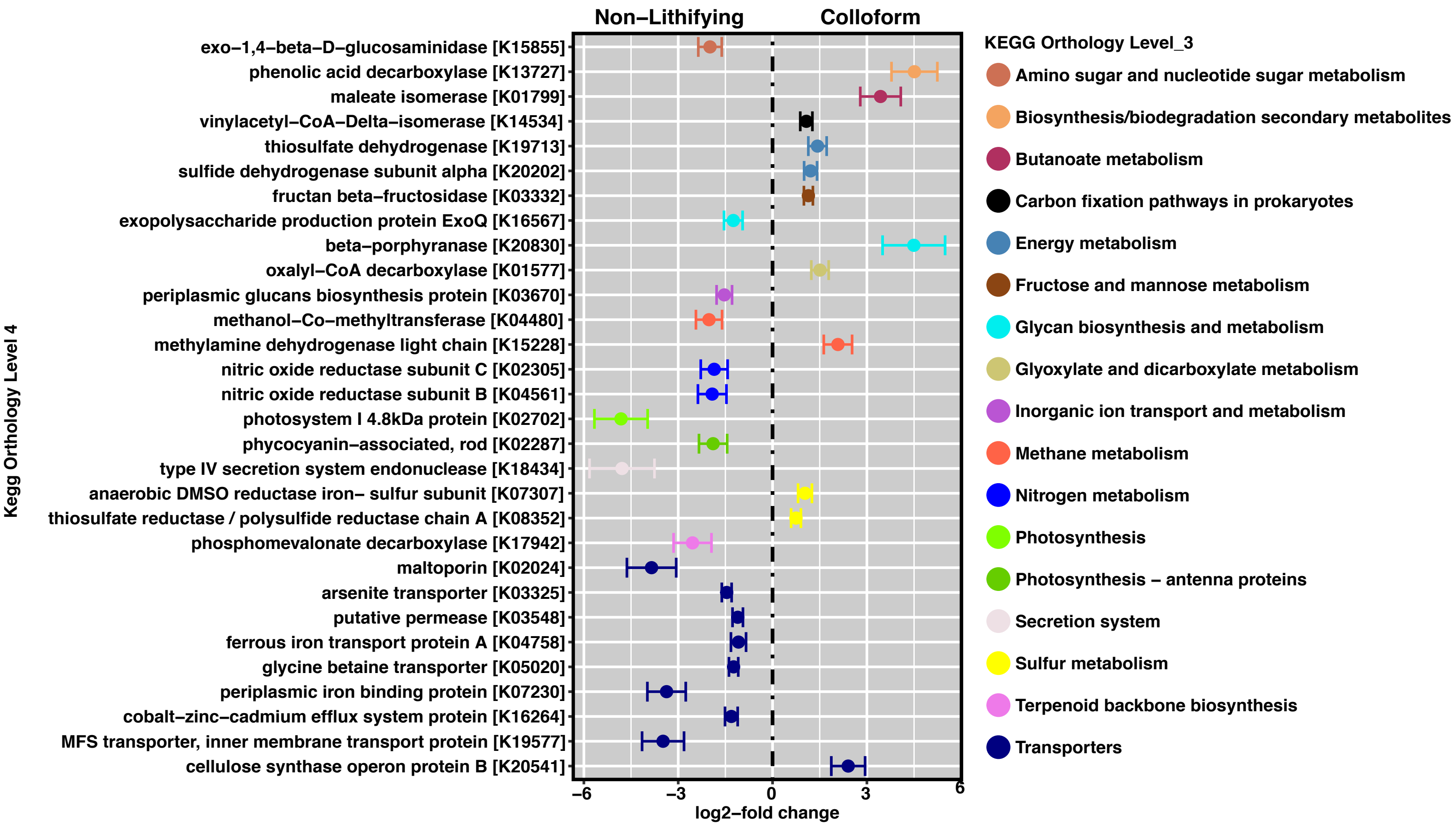

Supplemental Figure S5. Pairwise comparison of differentially abundant genes between non-lithifying and colloform mat types. Representative differentially abundant genes ($p < 0.001$) colored by their respective pathways. A positive log2-fold change designates differential abundance in the colloform mats and a negative log2-fold change designates differential abundance within the non-lithifying mat types. Lines indicate the standard error of the mean.

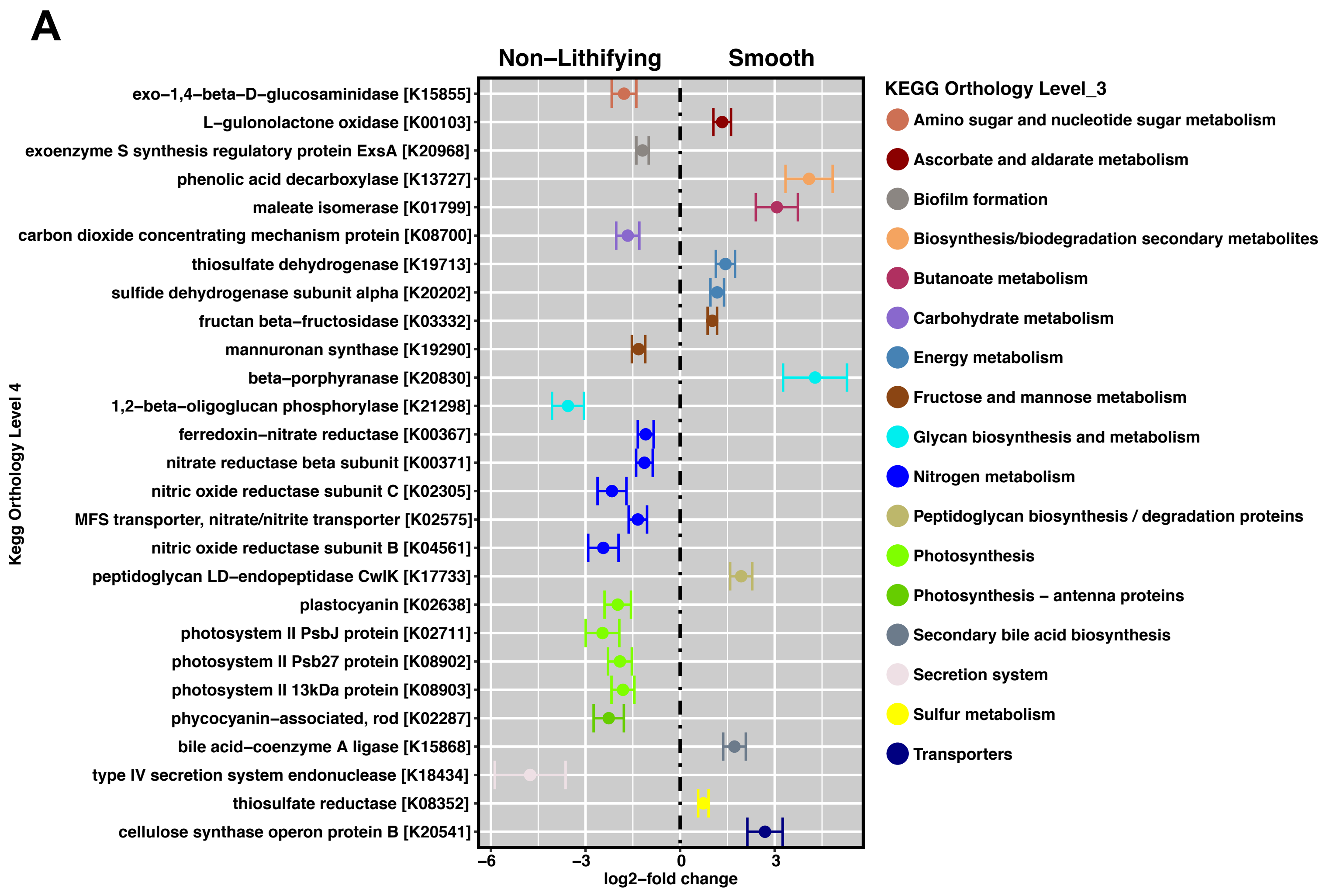

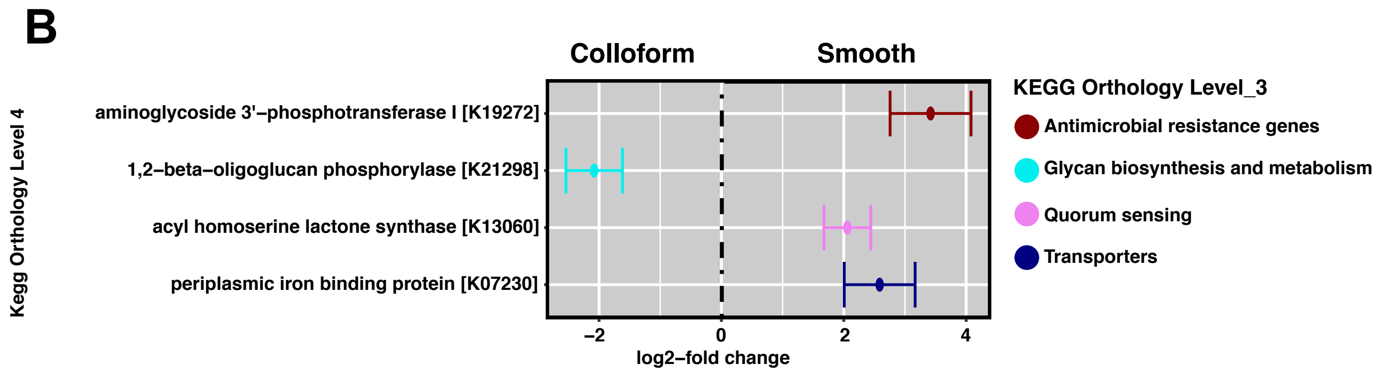

Supplemental Figure S6. Pairwise comparison of differentially abundant genes between non-lithifying and lithifying mats. Representative differentially abundant genes ($p < 0.001$) colored by their respective pathways. A positive log2-fold change designates differential abundance on the mat type on the right and a negative log2-fold change designates differential abundance within the mat type on the left. Lines indicate the standard error of the mean.

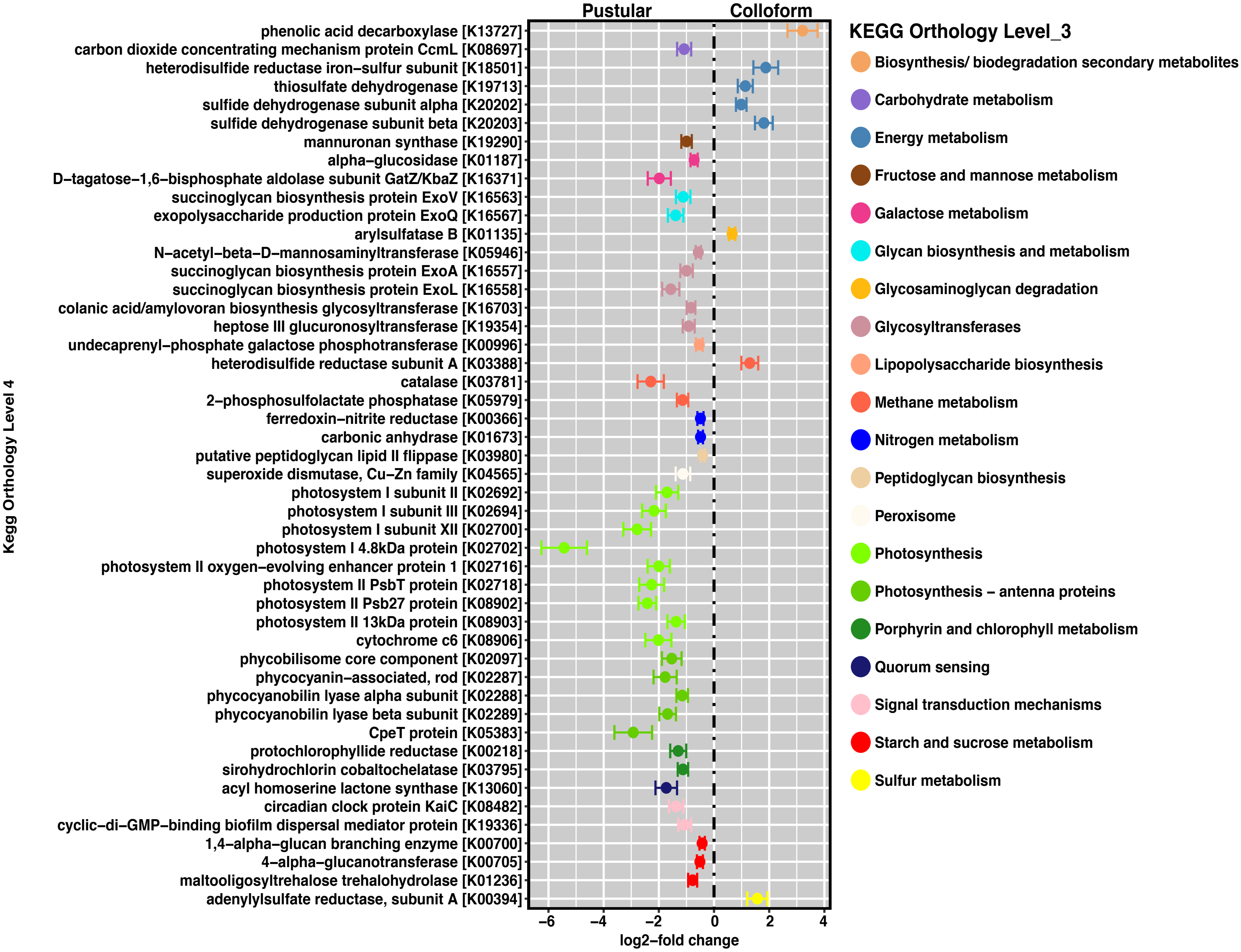

Supplemental Figure S7. Pairwise comparison of differentially abundant genes between pustular and colloform stromatolite-forming mats. Representative differentially abundant genes ($p < 0.001$) colored by their respective pathways. A positive log2-fold change designates differential abundance in the colloform mat type and a negative log2-fold change designates differential abundance within the pustular mat type. Lines indicate the standard error of the mean.

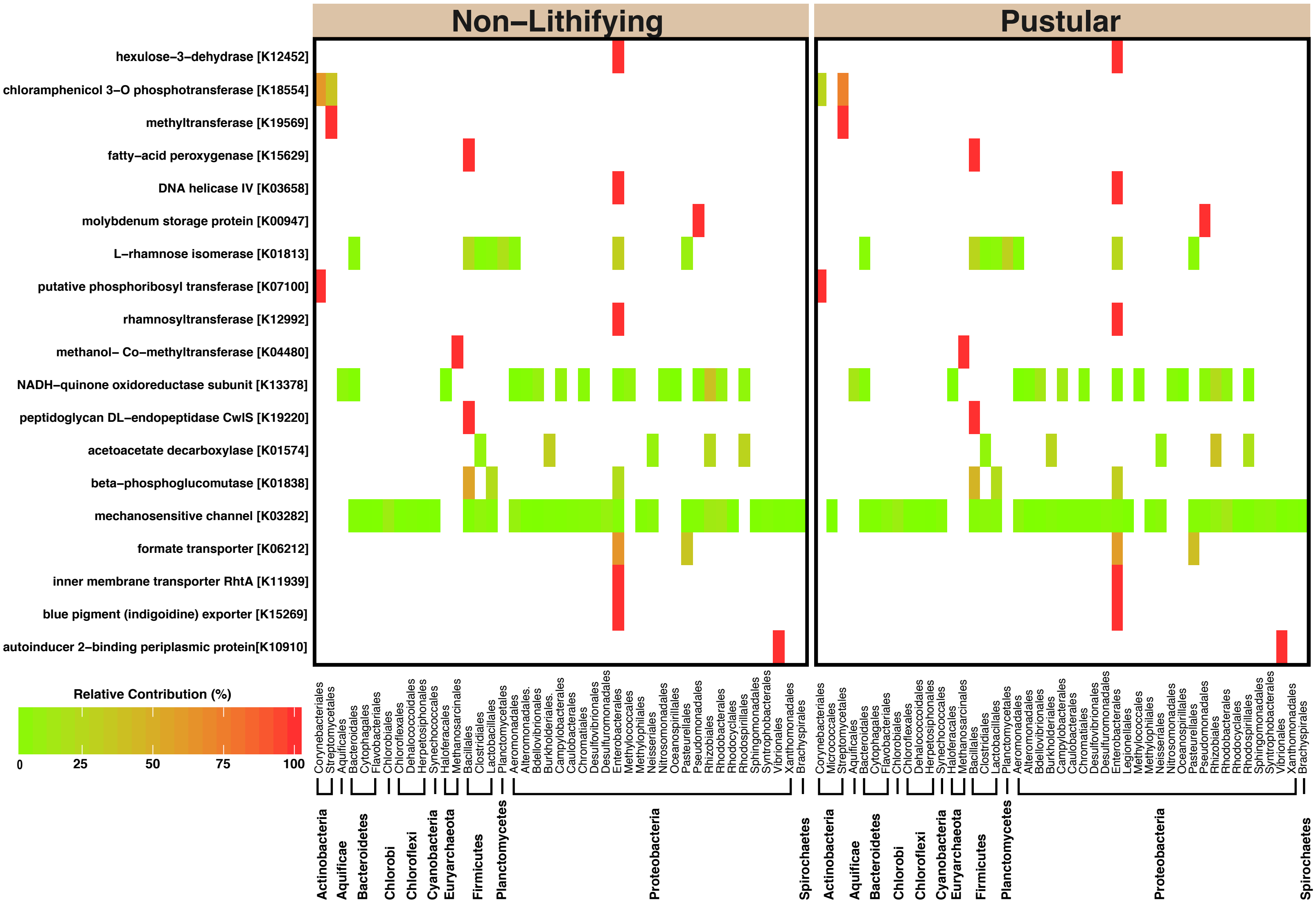

Supplemental Figure S8. Taxa associated with selected functional pathways. Heatmap showing the relative contribution of each taxon with respect to the differentially abundant genes within the non-lithifying mats and the pustular stromatolite-forming mats.

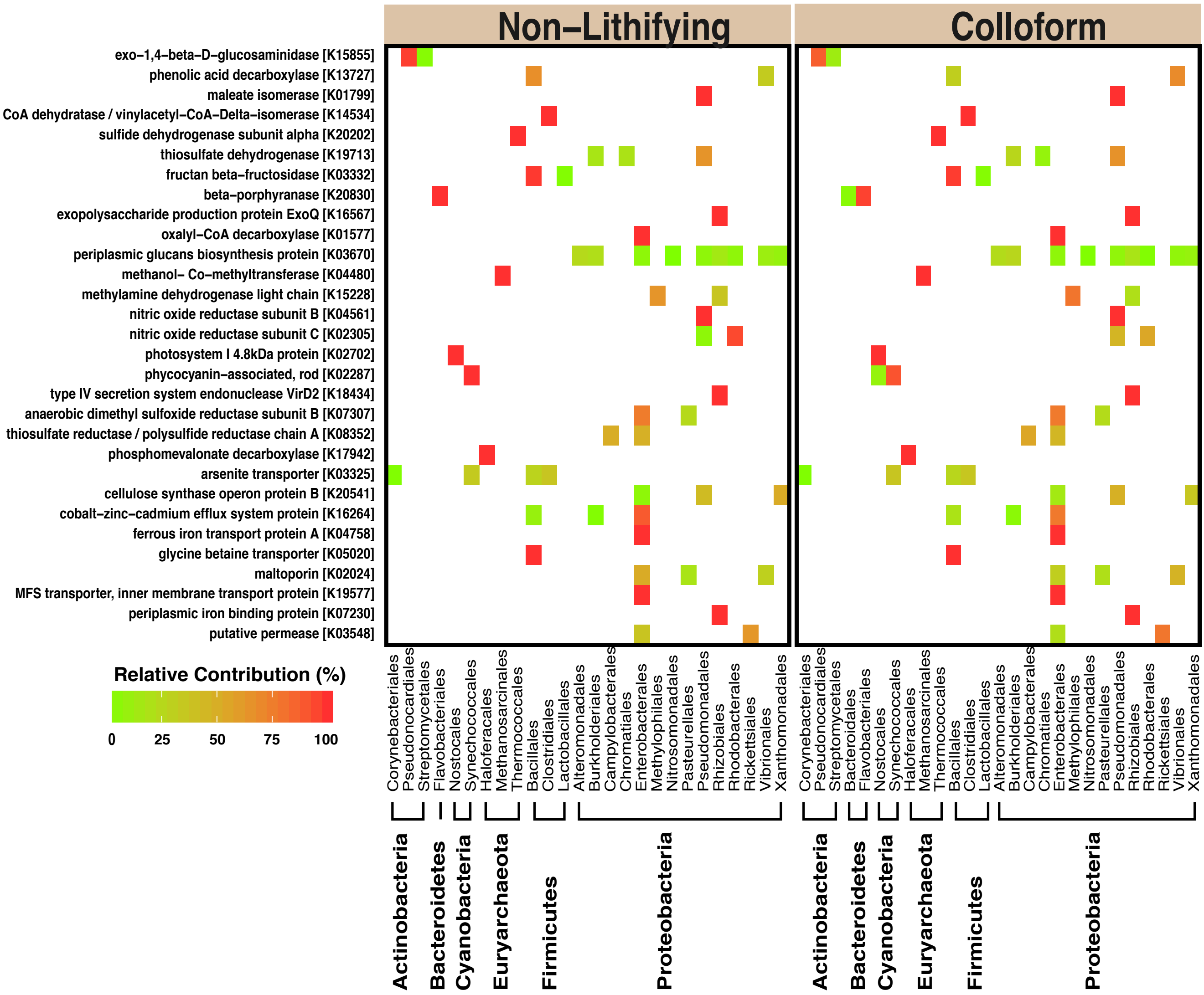

Supplemental Figure S9. Taxa associated with selected functional pathways. Heatmap showing the relative contribution of each taxon with respect to the differentially abundant genes within the non-lithifying mats and the colloform stromatolite-forming mats.

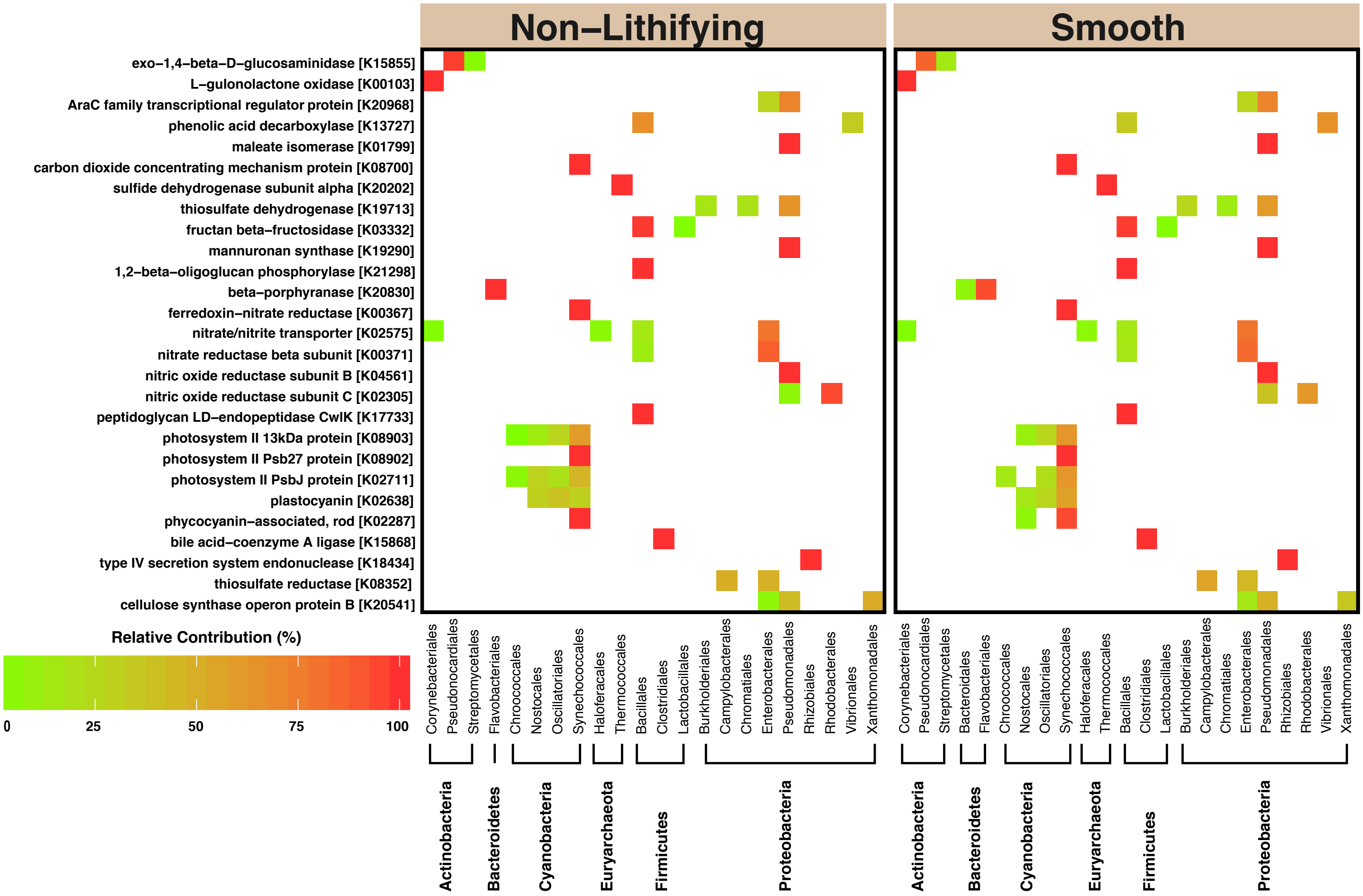

Supplemental Figure S10. Taxa associated with selected functional pathways. Heatmap showing the relative contribution of each taxon with respect to the differentially abundant genes within the non-lithifying and the smooth stromatolite-forming mats.

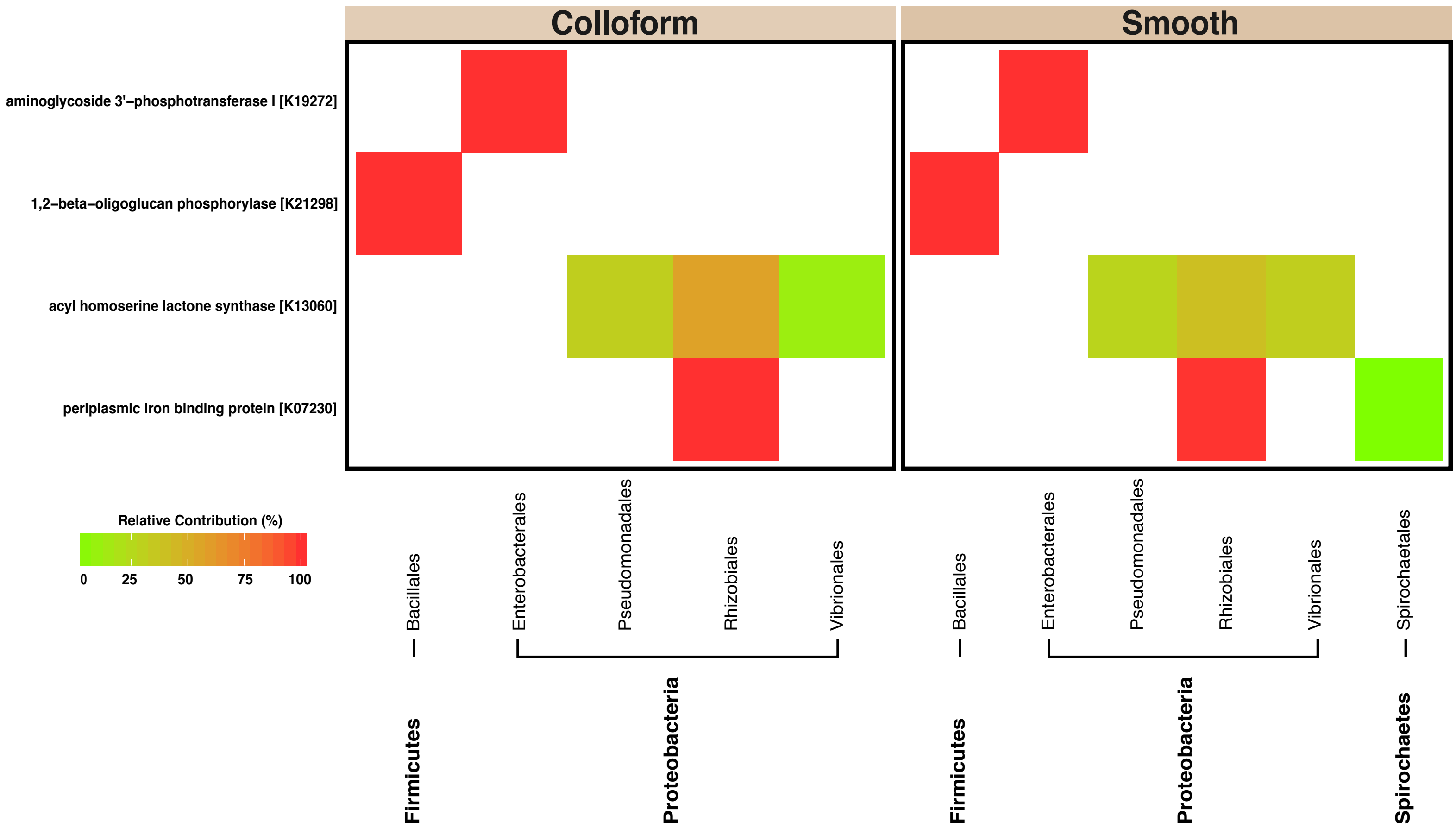

Supplemental Figure S11. Taxa associated with selected functional pathways. Heatmap showing the relative contribution of each taxon with respect to the differentially abundant genes within the colloform and the smooth stromatolite-forming mats.

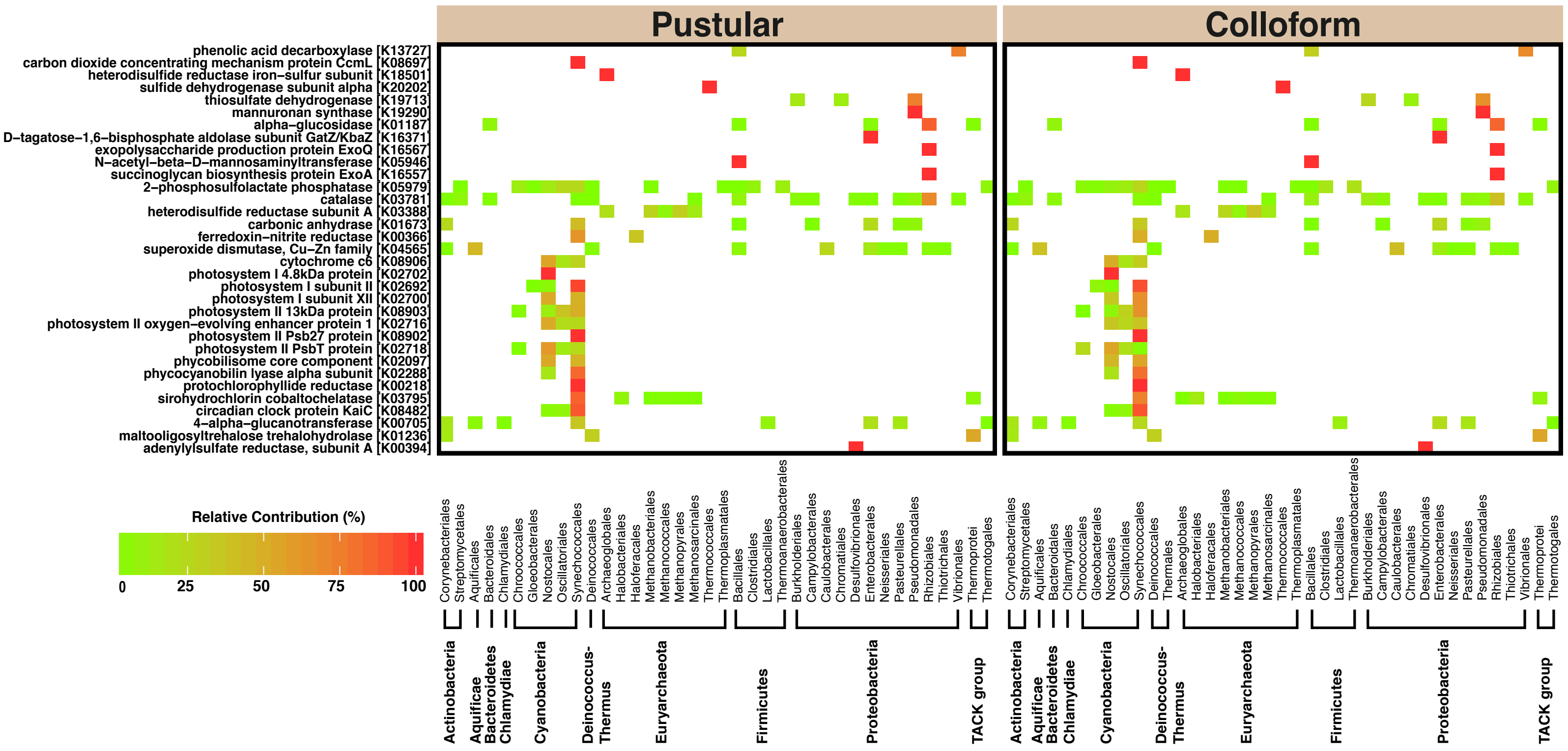

Supplemental Figure S12. Taxa associated with selected functional pathways. Heatmap showing the relative contribution of each taxon with respect to the differentially abundant genes within the pustular and the colloform stromatolite-forming mats.
